# A transcriptional cofactor regulatory network for the *C. elegans* intestine

**DOI:** 10.1093/g3journal/jkad096

**Published:** 2023-04-29

**Authors:** Brent B Horowitz, Shivani Nanda, Albertha J M Walhout

**Affiliations:** Department of Systems Biology, University of Massachusetts Chan Medical School, 368 Plantation Street, Albert Sherman Center, Worcester, MA 01605, USA; Department of Systems Biology, University of Massachusetts Chan Medical School, 368 Plantation Street, Albert Sherman Center, Worcester, MA 01605, USA; Department of Systems Biology, University of Massachusetts Chan Medical School, 368 Plantation Street, Albert Sherman Center, Worcester, MA 01605, USA

**Keywords:** *C. elegans*, gene expression, chromatin modifier, cofactor, intestine, gene regulatory network, RNAi collection

## Abstract

Chromatin modifiers and transcriptional cofactors (collectively referred to as CFs) work with DNA-binding transcription factors (TFs) to regulate gene expression. In multicellular eukaryotes, distinct tissues each execute their own gene expression program for accurate differentiation and subsequent functionality. While the function of TFs in differential gene expression has been studied in detail in many systems, the contribution of CFs has remained less explored. Here, we uncovered the contributions of CFs to gene regulation in the *Caenorhabditis elegans* intestine. We first annotated 366 CFs encoded by the *C. elegans* genome and assembled a library of 335 RNAi clones. Using this library, we analyzed the effects of individually depleting these CFs on the expression of 19 fluorescent transcriptional reporters in the intestine and identified 216 regulatory interactions. We found that different CFs regulate different promoters, and that both essential and intestinally expressed CFs have the greatest effects on promoter activity. We did not find all members of CF complexes acting on the same set of reporters but instead found diversity in the promoter targets of each complex component. Finally, we found that previously identified activation mechanisms for the *acdh-1* promoter use different CFs and TFs. Overall, we demonstrate that CFs function specifically rather than ubiquitously at intestinal promoters and provide an RNAi resource for reverse genetic screens.

## Introduction

Proper spatiotemporal gene expression is necessary for organismal growth and development, as well as maintaining cellular homeostasis and mounting stress responses. Gene expression depends on chromatin state and is governed on many levels. First and foremost, gene expression is regulated by transcription factors (TFs) that bind DNA elements located in gene promoters and enhancers and either activate or repress transcription. Most TFs regulate multiple genes, and each gene may be controlled by several TFs. Such complex relationships among genes and TFs can be captured in gene regulatory networks (GRNs) (Walhout 2006; Rogers and Bulyk 2018). Expression profiling by RNA-seq has enabled the identification of gene expression programs for many individual tissues and cells in a variety of organisms. However, the underlying GRNs that direct those programs are not well understood.

While TFs are often considered the primary drivers of specific gene expression programs, transcriptional cofactors (CFs) also play central roles in regulating transcription and, therefore, in establishing GRNs. CFs, which do not bind DNA directly, fall into two classes. The first class of CFs interact with TFs and RNA polymerase II at the promoter and regulate polymerase activity during several steps in the transcription cycle, including initiation, pausing, elongation and termination (Haberle and Stark 2018; Cramer 2019). The second class are chromatin modifiers that covalently modify histones or remodel nucleosomes to change chromatin structure and accessibility for TFs and RNA polymerase II (Bannister and Kouzarides 2011; Talbert *et al*. 2019).

CFs often function within multiprotein complexes that frequently show modularity. Smaller complexes tend to have core members all of which are essential for complex function and regulate the expression of similar gene sets, i.e. loss or perturbation of each disrupts complex functionality (Kemmeren *et al*. 2014). Larger complexes, such as the thirty-member Mediator complex, are often composed of submodules, each responsible for regulating the expression of subsets of genes (Lenstra *et al*. 2011; Kemmeren *et al*. 2014; El Khattabi *et al*. 2019). Additionally, submodules of some CF complexes, such as SAGA and Mediator, can work both together and independently of each other to regulate gene expression (Anandhakumar *et al*. 2016; Li *et al*. 2017). Further, subunits are sometimes shared between complexes. For example, the NuA4 histone acetyltransferase (HAT) complex and the histone depositing complex SWR1-C share four components (Lu *et al*. 2009). This complexity makes it difficult to study how CFs influence the expression of individual genes, and how interactions with other CF complexes, TFs and the basal transcriptional machinery affect gene regulation.

Chromatin modifying enzymes and the epigenetic marks placed by some of these enzymes are found at specific positions throughout the genome, yet not every gene associated with these factors changes expression upon their depletion (Lenstra *et al*. 2011; Venters *et al*. 2011; Weiner *et al*. 2012). This indicates that CFs have specific effects on gene expression that cannot be solely explained by binding profiles. How then does this specificity arise and what conditions lead to CF-specific gene regulation? Recent work has shown that certain CF complexes that were once thought to universally regulate gene expression are instead specific for certain types of promoters and enhancers (Haberle *et al*. 2019; Neumayr *et al*. 2022). Additionally, some chromatin remodeler complexes specify spatiotemporal gene expression throughout development, while others are used to regulate housekeeping genes that have stable developmental expression (Hendy *et al*. 2022). This indicates that nucleosome/chromatin structure may be different at these regulatory regions requiring different types of remodeler activities for gene activation and/or repression.

Delineating how CFs work together and with TFs to establish gene specificity in various contexts is important for understanding GRNs and how these influence growth, development, homeostasis, and cellular reactions to changing environments. Strategies for delineating GRNs have fallen into two categories: regulator (or protein)-centered approaches and gene-centered approaches (Johnson *et al*. 2007; McIsaac *et al*. 2012; Araya *et al*. 2014; Kemmeren *et al*. 2014; Fuxman Bass *et al*. 2015; MacNeil *et al*. 2015; Fuxman Bass *et al*. 2016). Regulator-centered approaches identify the global complement of genes bound and/or regulated by individual factors while gene-centered approaches focus on a specific gene and its regulatory sequences to identify the complement of regulators that bind or regulate the activity of that gene.

Determining GRNs for specific tissues or cells has been challenging. Single-cell RNA-seq has enabled gene expression measurement at the level of individual cells, and these measurements can be grouped into different cell and tissue types (Dixit *et al*. 2016). Other studies have inferred tissue-specific GRNs from a combination of gene expression data and protein-protein interactions (Sonawane *et al*. 2017). However, it is only just becoming feasible to systematically perturb each TF and measure tissue-specific gene expression changes at the same resolution in order to elucidate function (Adamson *et al*. 2016; Dixit *et al*. 2016; Liu *et al*. 2022).

The nematode *Caenorhabditis elegans* provides a powerful model to delineate GRNs for individual tissues. *C. elegans* has a transparent body, which allows the visualization of fluorescent proteins expressed under the control of specific gene promoters in vivo (Chalfie *et al*. 1994). With *C. elegans* transgenes, changes in fluorescent reporter expression in specific tissues upon gene knockdown can be monitored visually in living animals. This approach was used with a collection of 19 transgenic strains and comprehensive TF RNAi to delineate an in vivo activity-based GRN for the *C. elegans* intestine. This GRN contains 411 regulatory interactions driven by 177 TFs (MacNeil *et al*. 2015). TF knockdown mostly decreased reporter expression, indicating that TFs overall predominantly function as activators. One major insight from this study was that many of the regulatory effects of TF knockdown are likely indirect. Many TFs that showed activity in the screen were not found to physically interact with the promoters they regulate. Organizing the effects of different TFs on different promoters by nested effects modeling led to a hierarchical model in which TFs, directly or indirectly, regulate other TFs. Importantly, the TFs that were placed low in the hierarchy tended to physically interact with the promoters they regulate.

The *C. elegans* intestine is a highly metabolic tissue that functions not only as gut, but also performs mammalian liver-like functions (Kaletsky *et al*. 2018; Yilmaz *et al*. 2020). Since gene expression is frequently regulated by changes in metabolism and *vice versa* (Watson *et al*. 2015; Giese *et al*. 2019), a similar RNAi screen to that above was performed by depleting ∼1,500 metabolic genes and testing their effect on the activity of the same 19 promoters. This led to the identification of 1,251 regulatory interactions involving 512 metabolic genes (Bhattacharya *et al*. 2022). In contrast to TF knockdown, metabolic gene knockdown tended to increase reporter expression, indicating that metabolic perturbations mostly activate transcription. Interestingly, it was found that certain types of metabolism affect promoter activity over other types. For instance, many promoters were affected by perturbations in oxidative phosphorylation, while little to no effect was seen upon depletion of carbohydrate metabolism. Additional insights were gained through the identification of TFs that act downstream of some of these metabolic processes. Overall, the above results examining the interplay between TFs and metabolic enzymes in the regulation of gene expression suggest that in the *C. elegans* intestine, metabolic perturbations are commonly sensed by TFs that then function either directly or indirectly to regulate gene expression.

Since TFs work with non-DNA binding CFs to activate or repress their targets, we reasoned that additional information about the GRN in the *C. elegans* intestine could be obtained by asking globally how CFs influence promoter activity. While previous studies have individually deleted the entire complement of yeast CFs or degraded specific mammalian CFs and examined transcriptome-wide changes in gene expression (Lenstra *et al*. 2011; Haberle *et al*. 2019; Hendy *et al*. 2022; Neumayr *et al*. 2022), no study has comprehensively depleted CFs and examined the effects on a per gene basis. Here, we extend our gene-centered approach of using RNAi and specific promoters driving fluorescent reporters to screen a library of transcriptional CFs. We assembled a library of RNAi strains targeting most CFs encoded by the *C. elegans* genome and examined the effects of those CF knockdowns on the same 19 strains that were assessed in the previous two studies (MacNeil *et al*. 2015; Bhattacharya *et al*. 2022). We found that, while knockdown of most CFs had no effect on the 19 promoter reporters, depletion of a few CFs affected many promoters. Additionally, we assessed which CFs function in three independent activation mechanisms of a single promoter reporter, *Pacdh-1::GFP.* This study provides a basis for better understanding how CFs function within the *C. elegans* intestine as well as an RNAi resource for future studies.

## Materials and methods

### CF annotations

Previously, a preliminary set of CF predictions used the following categories: histone methyltransferases, histone demethylases, HATs, histone deacetylases (HDACs), TATA-binding protein (TBP) associated factors, Mediator components, and any gene encoding a protein with a plant homeodomain, chromodomain, or bromodomain (Reece-Hoyes *et al*. 2013). There was also a set of literature-defined CFs in an “other” category. We eliminated genes previously annotated as CFs that had no annotated CF function but only encoded enzymes with a particular enzymatic function (for example, a cytosolic methyltransferase with no histone targets) (Reece-Hoyes *et al*. 2005; Reece-Hoyes *et al*. 2013). We also removed reannotated pseudogenes and dead genes. We then added categories for chromatin remodelers, histone kinases, histone phosphatases, histone ubiquitinases, coregulators, RNA polymerase II-associated factors, and Tudor-domain containing proteins. Using WormBase version WS284, we searched for additional genes matching those categories Gene Ontology and InterPro protein motif terms (Ashburner *et al*. 2000; Blum *et al*. 2021). Additional factors were found through homology to yeast and human CFs. We also revised the literature-defined CFs in the other category, including adding newly annotated CFs acting in dosage compensation (Brejc *et al*. 2017). Several other CF annotations have been compiled (Cui and Han 2007; Tursun *et al*. 2011). However, these included sequence-specific TFs, proteins with chromatin-associated functions that do not involve transcriptional regulation, and/or histones and their variants. Since we aimed to specifically focus on CFs that do not directly bind DNA, we did not include histones, histone variants, nor sequence-specific TFs. In total, we annotated 366 *C. elegans* CFs (Supplementary Table 1).

### Essentiality enrichment analysis

*C. elegans* phenotypes were obtained using the SimpleMine tool in WormBase version WS284 (http://wormbase.org). A total of 19,987 protein-coding genes were included in the analysis. We considered essential genes as any gene with at least one of the following phenotypes: lethal, larval lethal, larval arrest, embryonic lethal, embryonic arrest, or sterile. Hypergeometric distribution was used to determine which CF categories or complexes are enriched for essential phenotypes (Supplementary Table 2).

### Tissue expression of CFs

We previously used a single-cell RNA-seq dataset to derive tissue expression of *C. elegans* genes at the second larval stage (L2) (Yilmaz *et al*. 2020). Here, we used this tissue gene expression data to evaluate in which tissues CFs are expressed.

### CF RNAi library

To construct the CF RNAi library, we obtained all available RNAi clones in from either the ORFeome or the Ahringer library (Kamath *et al*. 2003; Rual *et al*. 2004). We verified each clone by Sanger sequencing and if both clones for a given gene were correct, we only included the ORFeome clone. If a CF was not available in either library, we attempted to clone the ORF from newly synthesized cDNA (see below). The Gateway system was used to clone each cDNA into pDONR221, then subsequently into L4440-Dest-RNAi vector, followed by transformation into the RNAi-competent *E. coli* strain HT115(Walhout *et al*. 2000). All new clones were sequence verified. The final library was organized first by function, then alphabetically in 96-well plates; each plate also included vector control, GFP and mCherry RNAi clones, and blank wells (Supplementary Table 1). The library contains 335 RNAi clones: 186 from the ORFeome, 95 from the Ahringer library, and 54 new clones. RNAi clones for *tag-153* and *cbp-2* are not included in the library plates and were cloned separately. Genotyping primers and primers for the de novo clones are listed in Supplementary Table 6.

### *C. elegans* strains

*C. elegans* strains were maintained on nematode growth media (NGM) agar seeded with an *E. coli* OP50 diet as described, with some exceptions as noted below (Brenner 1974). The 19 transgenic promoter strains were described previously (Supplementary Table 3) (MacNeil *et al*. 2015). The *mthf-1(ww50); nhr-10(tm4695); Pacdh-1::GFP* strain (Giese *et al*. 2020) was maintained on NGM using soy peptone in place of bactopeptone containing 0.64 nM vitamin B12 (Sigma V2876).

### RNAi screening

RNAi screening was performed as previously described (Conte *et al*. 2015; MacNeil *et al*. 2015). Briefly, *E. coli* HT115 harboring individual CF RNAi plasmids were incubated overnight in 1 mL lysogeny broth (LB) + 50 µg/mL ampicillin + 10 µg/mL tetracycline in 96-deep-well plates at 37°C shaking at 200 rpm. The next day, 50 µL of overnight culture was transferred into 1 mL LB + 50 µg/mL ampicillin in 96-deep-well plates and incubated at 37°C at 200 rpm for 6 hours. Ten microliter of this culture was added to 96-well plates of NGM containing 50 µg/mL ampicillin and 2 mM isopropyl ß-D-thiogalactopyranoside (IPTG). Plates were dried and incubated overnight at room temperature. 20 to 40 synchronized L1 animals were added to the bacteria containing NGM agar plates and incubated at 20°C for 48 hours. Animals exposed to CF RNAi were visually scored for changes in intestinal fluorescence relative to vector control animals. Fluorescence changes in other tissues were not recorded. Each of the 19 strains was screened three times verses the entire RNAi library. Primary hits were considered as any interaction which occurred in two or three of the three replicates.

Primary hits were retested as above in duplicate, using 24-well NGM agar plates with 50 µL of bacterial culture in each well and 80–100 animals per well. After this step, hits were defined as any CF clone that scored positively in three of five combined replicates. Finally, RNAi was performed as above, using 6 cm plates, 100 µL of bacterial culture, and 150–200 animals per well. After 48 h of animal growth, animals were photographed under brightfield and either GFP or mCherry channels. Final hits were those that were confirmed in at least one of two photographed replicates. The *tag-153* RNAi strain was tested alongside the remainder of the library and is included in the interaction analyses. The *cbp-2* RNAi strain was only tested in the final experiment and is not included in interaction counts but is included in photos in the supplementary tables.

### Promoter enrichments

Hypergeometric distribution was used to determine CF categories or complexes that are enriched for either increases or decreases in fluorescence with each of the 19 transgenic promoter strains (Supplementary Table 5).

### qRT-PCR

*C. elegans* animals were grown as described above for RNAi screening using 10 cm NGM plates. After 48 hours of growth, animals were washed in M9 buffer and mRNA was extracted using the Direct-Zol RNA Miniprep Kit (Zymo Research R2050), including DNAse I treatment. cDNA was prepared using Oligo(dT) 12-18 Primer (Thermo Fisher 18418) and M-MuLV Reverse Transcriptase (NEB M0253). qPCR was performed in biological and technical triplicate using an Applied Biosystems QuantStudio 3 Real-Time PCR System with Fast SYBR Green Master Mix (Thermo Fisher 4385617). Relative transcript abundance was calculated using the ΔΔCt method (Livak and Schmittgen 2001). Endogenous controls were *act-1* and *ama-1.* Primer sequences are provided in Supplementary Table 6.

### *Pacdh-1* activation mechanisms

For all three *Pacdh-1::GFP* activation conditions, we grew vector control, GFP RNAi, and CF or TF RNAi strains as above and plated as above. To test which TFs and CFs contribute to the regulation of the *acdh-1* promoter by vitamin B12 mechanism I (Bulcha *et al*. 2019), we incubated *Pacdh-1*::*GFP* animals on NGM agar containing 50 µg/mL ampicillin, 2 mM IPTG, 40 mM propionate, and 5 nM vitamin B12. To test which TFs and CFs contribute to the regulation of the *acdh-1* promoter by vitamin B12 mechanism II (Giese *et al*. 2020), we incubated *(wwls24[Pacdh-1::GFP, unc-119(+)]; nhr-10(tm4695); mthf-1(ww50)* animals on soy peptone-based NGM agar containing 50 µg/mL ampicillin, 2 mM IPTG, and 5 nM vitamin B12. To test which TFs and CFs contribute to the regulation of the *acdh-1* promoter by succinate dehydrogenase inhibition, referred to here as mechanism III, we incubated *sdha-2(tm1420); wwls24[Pacdh-1::GFP, unc-119(+)]* animals on NGM agar containing 50 µg/mL ampicillin, 2 mM IPTG, and 6.4 nM vitamin B12. For all three conditions, we photographed animals at 48 h post plating as above.

### Microscopy

All fluorescence microscopy was performed on a Nikon Eclipse 90i with a Nikon DS-FI1 color camera, using NIS Elements software. Animals were washed off the plates with M9 buffer and paralyzed in 1 mM levamisole in a microfuge tube. The suspended animals were briefly centrifuged, and 2 µL of paralyzed animals were placed on an agar pad. Animals were aligned with a hair pick and photographed with a 10X Nikon CFI Plan Fluor. GFP excitement range was 450–490 nm and emission was 500–550 nm, while mCherry excitation was from 528–553 nm and emission was 590–650 nm. Pseudocolorization was added linearly by NIS Elements, and images were not altered any further.

## Results

### *C. elegans* CF annotation

**Fig. 1.**
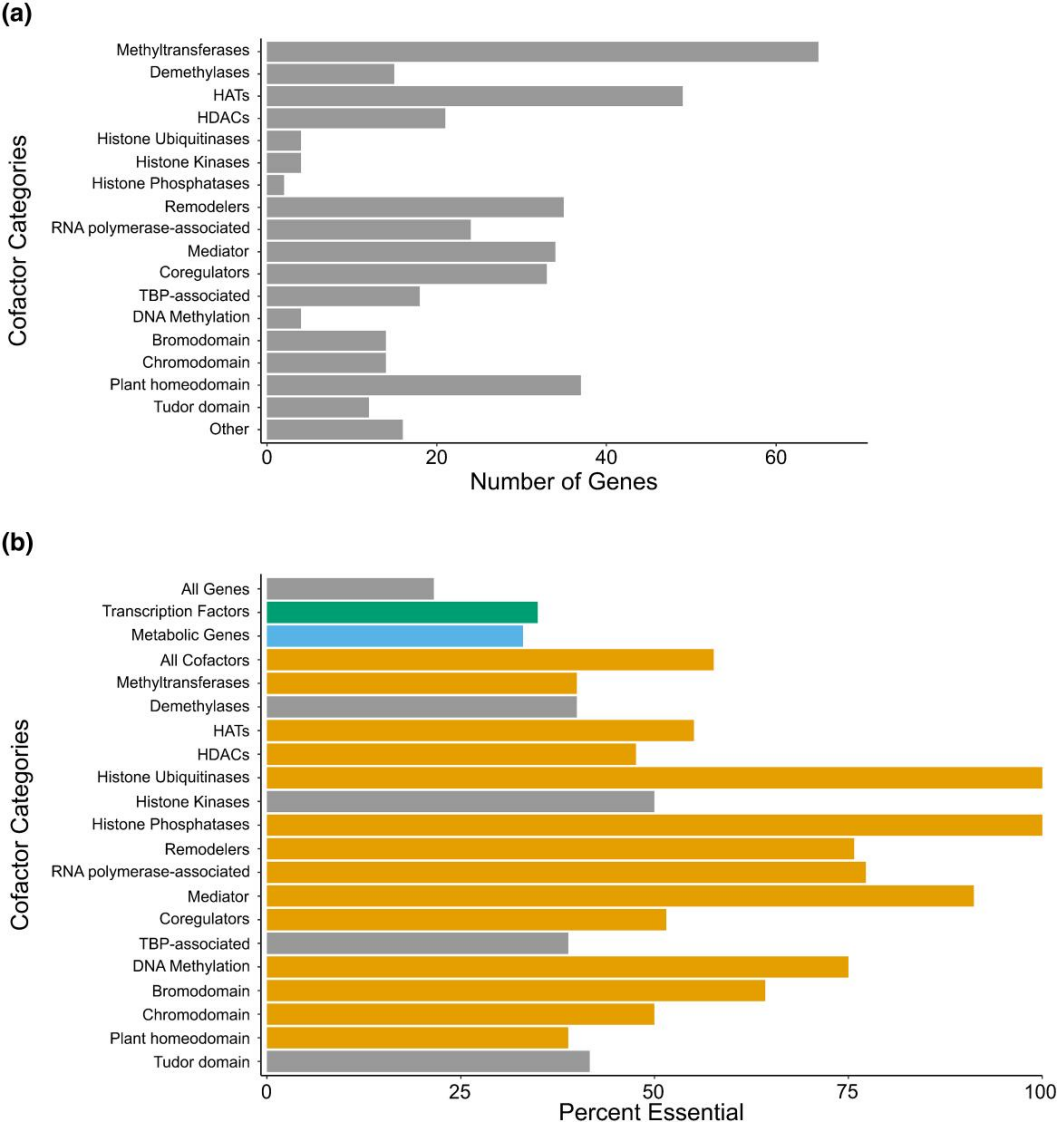
A compendium of *C. elegans* CFs. a) Classification of the 366 CFs encoded by the *C. elegans* genome. Some CFs are counted in multiple categories; for instance, SET-8 is counted twice because it is a methyltransferase and has a plant homeodomain. b) Percentage of each CF category that is annotated as essential (see Methods for essentiality definitions; *P*-values are listed in Supplementary Table 2). Grey bars indicate no enrichment for essentiality. Green and blue bars indicate an essentiality enrichment for TFs and metabolic genes, respectively. Orange bars indicate CF categories that are enriched for essentiality.

We previously annotated 228 *C. elegans* CFs (Reece-Hoyes *et al*. 2013). To extend this analysis and to comprehensively predict the complement of CFs encoded by the *C. elegans* genome, we used a combination of Gene Ontology, InterPro motifs, and sequence homology to known CFs in other species and classified these CFs by function or protein domain (Ashburner *et al*. 2000; Blum *et al*. 2021). Overall, we annotated 366 *C. elegans* CFs (Fig. 1a, Supplementary Table 1).

Many CFs function in multiprotein complexes. We used Gene Ontology annotations to identify ten *C. elegans* CF complexes based on sequence homology with well-studied CFs in other organisms. These CF complexes are predicted to be comprised of three to 31 proteins (Table 1). When we compared the composition of the complexes to their human homologs, we found that we were able to identify all or nearly all *C. elegans* orthologs for each complex. The one exception was the SAGA complex where we were only able to identify half of the components (9/18). Some CFs are known or predicted to be present in multiple CF complexes. For example, EKL-4 is a component of both NuA4 and SWR1 complexes (Lu *et al*. 2009). For the Mediator complex, we used existing annotations of homology, since many Mediator complex components have limited sequence homology among species but have high structural conservation across metazoa (Bourbon 2008; Cai *et al*. 2009).

**Table 1.** *C. elegans* CF complexes.

CF Complex	*C. elegans* Complex Components	Human Ortholog	Human Orthologs Not Found
CAF-1	*chaf-1, chaf-2, rba-1*	*CHAF1A, CHAF1B, CHAF1C*	
CCR4-NOT	*ccf-1, ccr-4, let-711, ntl-2, ntl-3, ntl-4, ntl-9, ntl-11, tag-153*	*CAF1, CCR4, CNOT1, CNOT2, CNOT3, CNOT4, CNOT9, CNOT11, CNOT2*	*CNOT10*
DRM	*dpl-1* * *, efl-1* * *, lin-9* * *, lin-35, lin-37* * *, lin-52, lin-53, lin-54* *	*DP1, E2F2, LIN9, RBL2, LIN37, LIN52, RBBP7, LIN-54*	
Mediator	*cdk-4, cdk-8, cic-1* * *, dpy-22, dyf-18, let-19, let-49, lin-25* * *, mdt-4, mdt-6* * *, mdt-8, mdt-10, mdt-11, mdt-15, mdt-17, mdt-18, mdt-19, mdt-20, mdt-21, mdt-22, mdt-27, mdt-29, mdt-31, R09F10.3* * *, rgr-1, sna-1, sop-3, sur-2*	*CDK4, CDK19, CCNC, MED12L, CDK7, MED13L, MED7, MED24, MED4, MED6, MED8, MED10, MED15, MED17, MED18, MED19, MED20, MED21, MED22, MED27, MED29, MED31, MED27, MED14, MED6, MED1, MED23*	*MED16, MED25*
NuA4	*B0025.4, cec-7, ekl-4, epc-1, F59E12.1, gfl-1, ing-3, mrg-1, mys-1, ruvb-1, ruvb-2, swsn-6* * *, trr-1, Y43H11AL.1, ZK1127.3*	*MEAF6, MORF4L1, DMAP1, EPC1, BRD8, YEATS4, ING3, MORF4L1, KAT5, RUVBL1, RUVBL2, ACTL6B, TRRAP, ING3, MRGBP*	*MEAF6*
NuRD	*dcp-66, egl-27, hda-1, let-418, lin-40*	*GATAD2B, RERE, HDAC2, CHD3, MTA3*	*MBD2*
SAGA	*ada-2, pcaf-1, T22B7.4, taf-6.1, taf-6.2* * *, taf-9, taf-10, taf-12, trr-1, Y17G9B.8* *	*TADA2B, KAT2B* , *SGF29, TAF6, TAF6, TAF9B, TAF10, TAF12, TRRAP, SGF29*	*ATXN7, ATXN7L3, ENY2, SF3B3, SF3B5, SUPT7L, SUPT20H, TADA1, TADA3, USP22*
Set1C/COMPASS	*ash-2, cfp-1, dpy-30, hcf-1, rbbp-5, set-2, swd-2.1, swd-2.2, wdr-5.1*	*ASH2L, CXXC1, DPY30, HCFC1, RBBP5, SETD1A, WDR82, WDR82, WDR5*	
SWI/SNF/BAF	*C52B9.8, dpff-1, ham-3, let-526, snfc-5, swsn-1, swsn-2.2, swsn-3, swsn-4, swsn-6* * *, swsn-7, ZK973.9*	*DPF2, SMARCD3, ARID1A, SMARCB1, SMARCC2, SMARCD1, SMARCE1 SMARCA2, ACTL6B, ARID2, SS18*	
SWR1	*arp-6* * *, C17E4.6, ekl-4, F13C5.2* * *, ruvb-1, ruvb-2, ssl-1, zhit-1*	*ACTR6, VPS72, DMAP1, BRD8, RUVBL1, RUVBL2, EP400, ZNHIT1*	

Names of the CF complexes are in the first column. Gene names are in the second column. Human orthologs are in the third column in the same order as the *C. elegans* gens in the second column. The fourth column contains any other complex members in humans we did not find in *C. elegans.*

indicates not included in the CF RNAi library.

### CFs are enriched for essentiality

Previous work has demonstrated that *C. elegans* CFs often genetically interact with many other genes and pathways (Lehner *et al*. 2006). We therefore next asked whether *C. elegans* CFs tend to be essential for viability. We first used publicly available data mined from WormBase version WS284 to comprehensively define essential *C. elegans* genes (Lee *et al*. 2018). We then compared the percentage of genes that are essential among all *C. elegans* genes with the percentage of CF, TF, and metabolic genes, and found all three of these gene categories and many CF categories are significantly enriched for essentiality (Fig. 1b, Supplementary Table 2). While methyltransferases, HATs, HDACs, histone ubiquitinases, histone phosphatases, remodelers, RNA-polymerase II-associated factors, Mediator components, DNA methylation enzymes and bromodomain-containing, chromodomain-containing, and plant homeodomain-containing proteins are more essential than random gene sets; demethylases, histone kinases, TBP-associated factors, and proteins harboring Tudor domains are not.

### A CF RNAi library resource

**Fig. 2.**
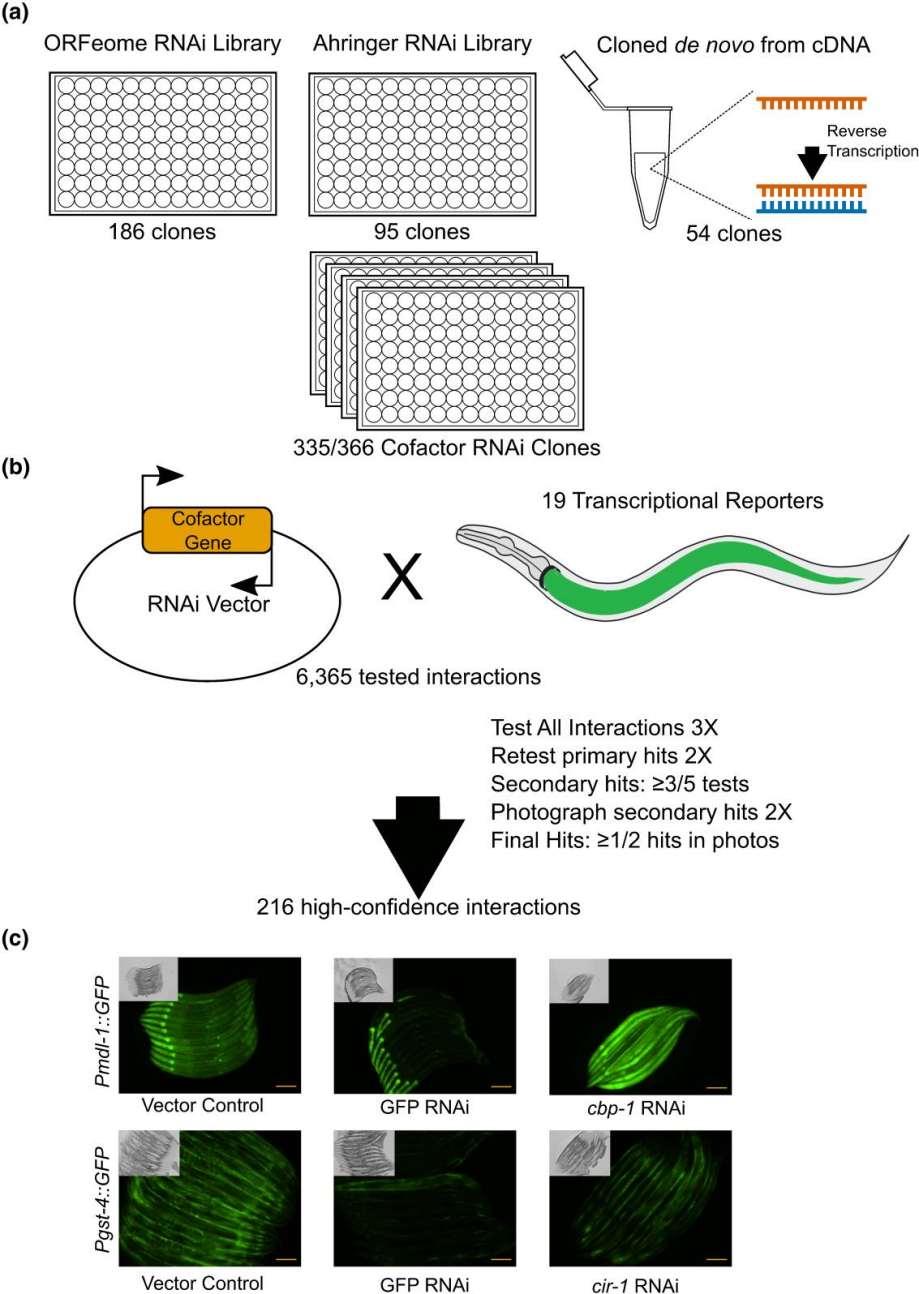
CF RNAi library assembly and RNAi screens. a) Assembly of the CF RNAi library from the ORFeome and Ahringer RNAi libraries as well as clones generated de novo. All clones are arrayed in four 96-well plates by function. Each plate contains vector controls as well as GFP and mCherry RNAi clones. b) Diagram of the RNAi screening strategy. The primary screen was performed in triplicate and all hits were retested in duplicate. Any hits found in 3/5 tests were photographed twice; final hits were those that retested in at least one photograph. c) Examples of CF RNAi changing intestinal fluorescence with two of the 19 promoter reporters. Scale bar = 100 µM.

RNAi-by-feeding is a useful tool to examine phenotypes caused by gene knockdown in *C. elegans.* Previous work has established genome-wide RNAi libraries that contained RNAi clones for many CFs (Fraser *et al*. 2000; Kamath *et al*. 2003; Rual *et al*. 2004). In order to construct a comprehensive CF library, we assembled a library of RNAi strains targeting 335 of the 366 *C. elegans* annotated CFs (92%) (Fig. 2a, Supplementary Table 1). Of these, 186 clones were obtained from the ORFeome collection (Rual *et al*. 2004), 95 were retrieved from the Ahringer collection (Kamath *et al*. 2003), and 54 were generated de novo. This library is, to the best of our knowledge, the most comprehensive *C. elegans* CF RNAi resource (Fig. 1a, Supplementary Table 1).

### Uncovering regulatory promoter-CF interactions in the *C. elegans* intestine

To understand the regulatory effects of CFs on promoter activity, we performed a visual screen using 19 transcriptional reporter strains that express a fluorescent protein within the *C. elegans* intestine (MacNeil *et al*. 2015) (Supplementary Table 3). We exposed L1 animals from each of the 19 reporter strains to knockdown of each CF and visually monitored fluorescence changes in the intestine after 48 hours, when the animals reached the young adult stage. This primary screen was done three times. Visual screens are noisy and many of the regulatory interactions found in the primary screen were subtle. Therefore, we retested any CF RNAi that caused a change in intestinal fluorescence in two or more replicates in any one reporter strain twice more with all 19 reporter strains using larger plates and more animals. Finally, we captured images for any change in fluorescence that was observed in at least three of the five tests of a given strain. Our final dataset includes only those regulatory interactions confirmed by a captured image (Fig. 2b–c, Supplementary Figs. 1–19 in Supplementary File 1, Supplementary Table 4). Note that hits that are subtle yet consistent, such as *ntl-2* RNAi, which decreased intestinal fluorescence in the *Psbp-1::GFP* strain, were kept in the final dataset (Supplementary Fig. 16 in Supplementary File 1).

**Fig. 3.**
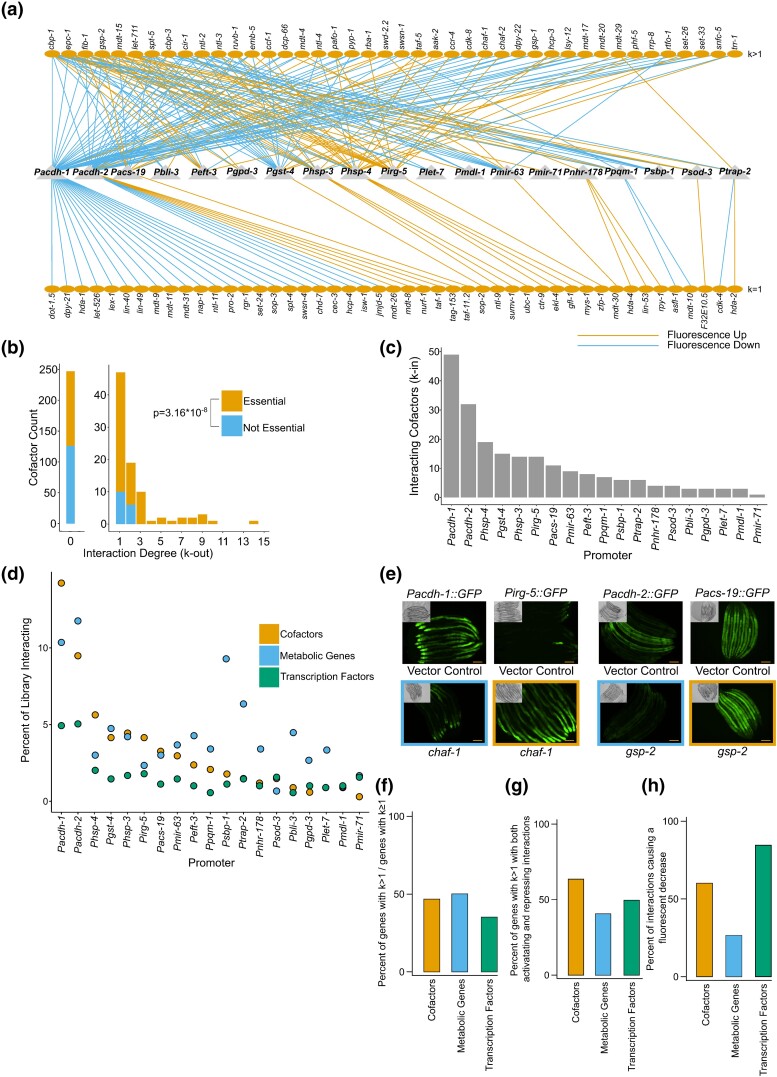
A CF regulatory network for the *C. elegans* intestine. a) Bipartite graph of CF-promoter interactions. Triangles represent the 19 promoters. Ovals represent CFs regulating these promoters in the *C. elegans* intestine. Orange edges represent increases in fluorescence (repressing interactions) and blue edges represent decreases in fluorescence (activating interactions). k indicates the out-degree, or number of interactions, of each CF. b) The k-out distribution. Orange bars represent essential CFs, blue bars represent CFs not annotated as essential. *P*-value was calculated using a Mann–Whitney test. c) In-degree (k-in) for each of the 19 promoter reporters, or the number of CFs affecting each promoter. d) Percentage of CFs, metabolic genes, and TFs (orange, blue, and green respectively) that interact with each promoter reporter, arranged by percent interacting CFs. e) Examples of CFs that both increase and decrease fluorescence in different promoter reporters. Scale bar = 100 µM. Colors as in A. f) Percentage of genes that affect multiple promoters, sorted by library. Colors as in B. g) Percentage of genes with multiple interactions that both increase and decrease fluorescence, sorted by library. Colors as in B. h) Percentage of interactions which showed fluorescence decrease, sorted by library. Colors as in B.

Altogether, we detected 216 regulatory interactions between 19 promoters and 89 CFs (3.3% of all tested interactions) (Fig. 3a). RNAi of more than half (61%) of these CFs showed a decrease in the expression of the intestinal reporter gene and therefore, these CFs potentially act as activators. Knockdown of the CBP/p300 HAT *cbp-1* changed the expression driven by 14 of the 19 promoters; ten reporters increased, and four reporters decreased in fluorescence. Therefore, *cbp-1* may function in both activation and repression of gene expression depending on the context. Alternatively, some of the observed regulatory interactions may be indirect. Depletion of most of the other CFs affected expression from only a small subset of the 19 promoters, indicating that these CFs mostly act in a gene-specific manner. We also observed an enrichment for interactions among essential CFs over non-essential CFs (Fig. 3b).

We next asked whether the CFs that affect any of the 19 promoters are expressed in the intestine or whether they are expressed in other tissues and therefore may exert their regulatory effects indirectly in a cell nonautonomous manner. We examined CF expression in a single-cell RNA-seq dataset that measured gene expression in seven tissues in the second larval (L2) stage and found that most *C. elegans* CFs are expressed in the animal's intestine (Supplementary Figure 20A in Supplementary File 1) (Cao *et al*. 2017). We found an enrichment of intestinally expressed CFs for regulatory interactions (Supplementary Figure 20A in Supplementary File 1), which could indicate that most uncovered interactions act cell autonomously. However, there were also enrichments for regulatory interactions in each other measured tissue (Supplementary Figure 20B–G in Supplementary File S1). Most CFs that affect promoter activity in our screen are expressed ubiquitously, leaving open the possibility that these effects are caused by CF activity in other RNAi-susceptible tissues.

To integrate our data with the TF GRN and metabolic MRN obtained in our previous screens, we then looked for commonalities between TFs, metabolic genes, and CFs in their effects on each promoter (MacNeil *et al*. 2015; Bhattacharya *et al*. 2022). Overall, we did not observe a uniform pattern. However, we did notice that the *Pacdh-1* and *Pacdh-2* promoters were regulated by more CFs, TFs, and metabolic genes than the other 17 promoters (Fig. 3c–d, Supplementary Figs. 1–2 in Supplementary File 1). Other promoters, such as *Psbp-1* and *Ptrap-2,* were affected by depletion of many metabolic genes, but relatively few TFs or CFs. By contrast, the *hsp-4* promoter was affected by depletion of a relatively large proportion of CFs but few TFs and metabolic genes (Fig. 3d).

Many CFs have been shown to have both activator and repressor functions in other organisms (Subramaniam *et al*. 1999; Villanueva *et al*. 2011; Hunt *et al*. 2022). Our data suggest that this may also be the case for *C. elegans*. In addition to *cbp-1* as discussed above, *chaf-1* RNAi decreased intestinal fluorescence of the *Pacdh-1::GFP* reporter, but increased GFP expression of the *Pirg-5::GFP* reporter (Fig. 3e). Similarly, *gsp-2* RNAi had opposite effects on *Pacdh-2::GFP* and *Pacs-19::GFP* (Fig. 3e). Altogether, 48% of CF knockdowns affected the activity of multiple promoters, which is similar to the 51% rate of metabolic gene RNAi but slightly higher than the 36% of TFs that affected multiple promoters (Fig. 3f). This could indicate broader roles for CFs in comparison with TFs, in line with previous studies showing CFs affect more genes on average than TFs (Kemmeren *et al*. 2014). Of the 42 CFs that exhibited a regulatory effect on at least two promoters, 27 both increased and decreased reporter expression when depleted (64%). This is modestly higher than the 50% of TFs and 41% of metabolic genes with multiple interactions that both activate and repress promoter activity (Fig. 3g) (MacNeil *et al*. 2015; Bhattacharya *et al*. 2022). CF knockdowns also caused a lower proportion of fluorescence decreases than TF knockdowns, but far more than metabolic gene knockdowns (Fig. 3h).

### CF complexes exhibit specificity and modularity

**Fig. 4.**
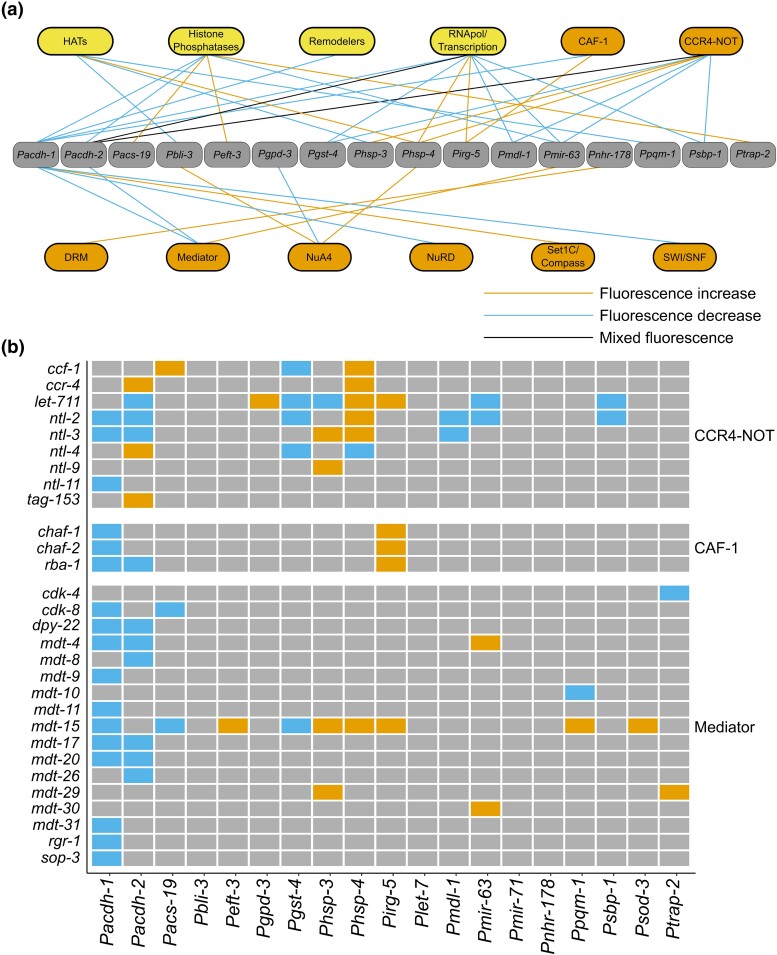
Enrichments and interaction profiles of CF complexes. a) Hypergeometric enrichments between CF categories (yellow) and complexes (orange) that regulate the promoter reporters. Categories, complexes, and promoters without enrichments are not included. b) Interaction profiles for CCR4-NOT, CAF-1, and Mediator complexes. Orange boxes represent increases in fluorescence (repressing interactions) and blue boxes represent decreases in fluorescence (activating interactions). For the Mediator complex, components that did not affect any of the promoters when knocked down by RNAi were not included. See also Supplementary Fig. 20.

Since CFs function in large complexes and many have overlapping functions, we asked whether CF complex components or CFs in the different functional categories affected the same set of promoters. Four CF categories and members of eight different complexes were statistically enriched for regulatory interactions with at least one promoter (Fig. 4a, Supplementary Table 5). For example, knockdown of several members of the CCR4-NOT and NuA4 complexes modulated GFP expression from the *Phsp-4* promoter (Fig. 4a–b, Supplementary Fig. 20 in File 1). Five of the CCR4-NOT complex members showed an increase in *Phsp-4::GFP* expression when knocked down, however, RNAi of one member, *ntl-4*, decreased reporter expression. Interestingly, this component is a ubiquitin-ligase that may play more of an overall regulatory function or may function outside of the CCR4-NOT complex as suggested previously (Halter *et al*. 2014). Additionally, each of the nine CCR4-NOT complex components affected at least one of the tested promoters, but no two component knockdowns had the same interaction profile (Fig. 4b). For the NuA4 complex, six components, *ekl-4, epc-1, gfl-1, mys-1, ruvb-1,* and *trr-1,* activated *Phsp-4::GFP* upon depletion (Supplementary Fig. 20 in File 1). While NuA4 shares some components with both the SAGA and SWR1-C complexes, of the components that activate *Phsp-4::GFP* upon knockdown, only *trr-1* is shared with SAGA complex while none are shared with SWR1-C (Table 1).

In our dataset, CF complexes did not act uniformly. The CAF-1 complex had the most uniform effects where two members, *chaf-1* and *chaf-2,* showed the same interaction profile: their knockdown decreased fluorescence with the *Pacdh-1::GFP* reporter, and increased fluorescence with the *Pirg-5::GFP* reporter (Fig. 4b). The third component, *rba-1,* showed the same effects on *Pirg-5 and Pacdh-1*, but its knockdown also decreased *Pacdh-2::GFP* expression. The nucleosome remodeling and deacetylase complex was also relatively uniform, with three components affecting *Pacdh-1::GFP* expression, while *dcp-66* had two additional regulatory interactions (Supplementary Fig. 20 in File 1). Additionally, three Mediator components, *dpy-22*, *mdt-17*, and *mdt-20,* have identical profiles showing specificity for the *Pacdh-1* and *2* promoters. Knockdown of the well-studied Mediator component *mdt-15* [(Yang *et al*. 2006), (Taubert *et al*. 2006; Taubert *et al*. 2008; Arda *et al*. 2010)] affected nine promoters and exhibited both activation and repression of promoter activity when knocked down. However, only five of those promoters were affected by depletion of other Mediator components (Fig. 4b, Supplementary Fig. 20 in File 1).

### Activation of *Pacdh-1* uses different TFs but common CFs

**Fig. 5.**
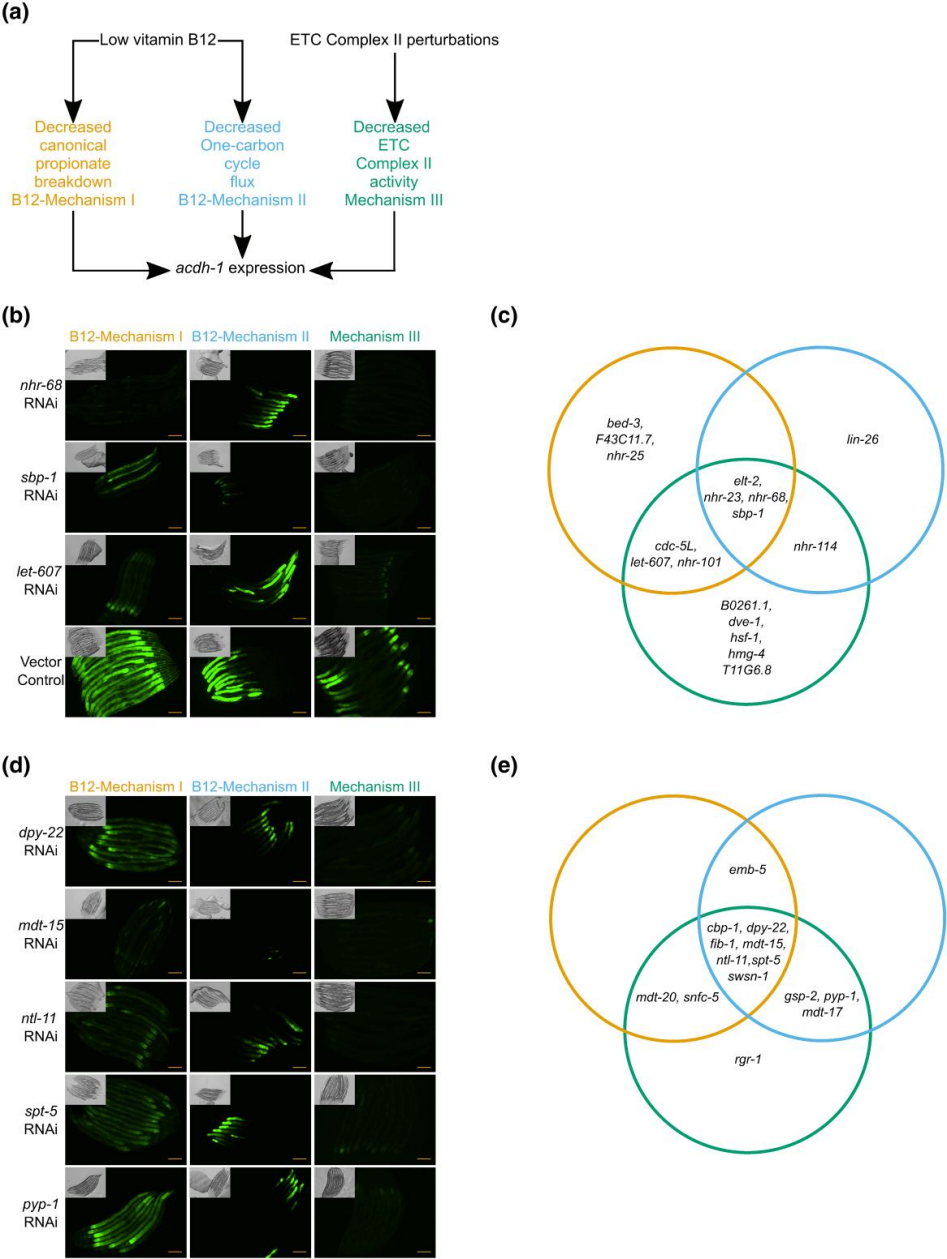
Different mechanisms that activate *Pacdh-1* use different TFs and CFs. a) Cartoon of the three mechanisms activating *Pacdh-1::GFP*. b) Examples of TFs that affect *Pacdh-1::GFP*. Photos of all TFs involved in at least one mechanism are provided in Supplementary Fig. 21. c) Venn diagram of all TFs involved in the three mechanisms. Colors are as in A. d) Examples of CFs involved in the regulation of *Pacdh-1::GFP*. Photos of all CFs that regulate *Pacdh-1::GFP* are provided in Supplementary Fig. 22. e) Venn diagram of all CFs involved in the three mechanisms. Colors are as in A. Scale bar = 100 µM.

The acyl-CoA dehydrogenase ACDH-1 is the first enzyme of the propionate shunt, an alternative breakdown pathway of this short chain fatty acid that is transcriptionally activated when the canonical, vitamin B12-dependent pathway is genetically or nutritionally perturbed (MacNeil *et al*. 2013; Watson *et al*. 2013; Watson *et al*. 2014; Watson *et al*. 2016; Bulcha *et al*. 2019). As shown above and in previous studies, *acdh-1* promoter activity is affected by the knockdown of many CFs, TFs, and metabolic genes (Fig. 3e) (MacNeil *et al*. 2015; Bhattacharya *et al*. 2022). We also previously found that the *acdh-1* promoter is activated in response to three specific metabolic perturbations. As mentioned above, bacterial diets low in vitamin B12 confer reduced flux through the canonical B12-dependent propionate breakdown pathway in *C. elegans* and activates *acdh-1* expression (Watson *et al*. 2016). Low dietary vitamin B12 also reduces flux through the Methionine/S-adenosylmethionine (Met/SAM) cycle, and mutations in Met/SAM cycle genes also increase *Pacdh-1::GFP* expression (Giese *et al*. 2020). Because both these activation mechanisms of *acdh-1* expression are caused by low vitamin B12; we have termed activation of *acdh-1* by excess propionate and low Met/SAM cycle activity as B12-Mechanism I and B12-Mechanism II, respectively (Giese *et al*. 2020). Perturbation of succinate dehydrogenase, which is also known as complex II of the electron transport chain, also activates *Pacdh-1::GFP* (Bhattacharya *et al*. 2022). To the best of our knowledge, low dietary vitamin B12 does not result in complex II dysfunction. Therefore, we refer to this mechanism of *acdh-1* activation as Mechanism III (Fig. 5a). While the TFs that mediate the response to each of these have been studied in some detail, the CFs involved remain unknown.

Previously, we identified forty-nine TFs that, when knocked down by RNAi, repress *Pacdh-1::GFP* under standard growth conditions (MacNeil *et al*. 2015). We asked which of these TFs participate in each of the three mechanisms of *Pacdh-1* activation by depleting each TF by RNAi in conditions that specifically assess each activation mechanism. We found that four TFs, *elt-2*, *nhr-23*, *sbp-1*, and *nhr-68*, are involved in all three mechanisms, while others, such as *let-607*, appear to function in only one or two of the mechanisms (Fig. 5b–c, Supplementary Fig. 22 in Supplementary File 1). Because analyzing B12-Mechanism II uses an *nhr-10* mutant, we did not place it in our diagram, but we note that it is required for both B12-Mechanism I and Mechanism III (Supplementary Fig. 22 in Supplementary File 1).

We performed the same analysis for CFs that affected *Pacdh-1::GFP* expression, where we determined which of the CFs found to interact with this promoter in the primary screen contributed to each of the three activation mechanisms (Fig. 5d, Supplementary Fig. 23 in Supplementary File 1). Remarkably, in contrast to TFs, several CFs that were used for any of the activation mechanisms were involved in all three mechanisms of *Pacdh-1* activation (Fig. 5e). Of the fourteen CFs used by at least one mechanism, seven were used in all three. Interestingly, there were five components of the Mediator complex required for Mechanism III activity: *dpy-22/mdt-12*, *mdt-15*, *mdt-17*, *mdt-20*, and *rgr-1/mdt-14.* Several TFs that regulate *Pacdh-1*, including SBP-1 and NHR-10, physically interact with MDT-15 (Arda *et al*. 2010). These results indicate that different combinations of TFs and CFs function together to induce *acdh-1* expression in response to different metabolic perturbations. Further, the observation that not all TF and CF knockdowns that activate *acdh-1* under standard conditions were found to act in any of the three mechanisms indicates that there could be additional mechanisms of *acdh-1* induction.

### Three CBP/p300 paralogs with only partially overlapping domains all regulate promoter activity

**Fig. 6.**
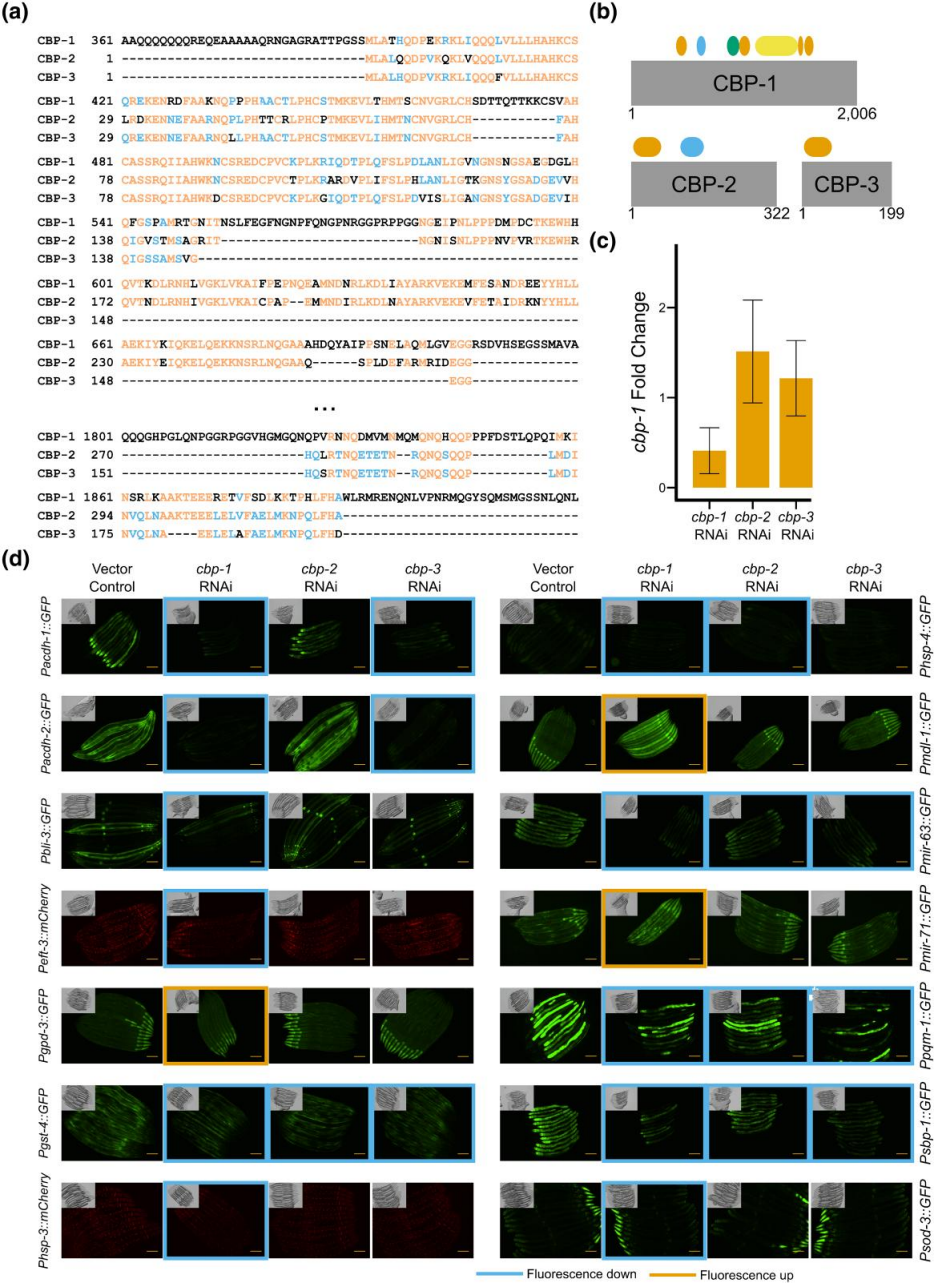
*cbp-1, cbp-2,* and *cbp-3* are functional genes. a) Sequence alignment of CBP-1, CBP-2, and CBP-3. Black letters are unique to one protein, blue letters are common to two proteins, and orange letters are common to all three proteins. Portions of *cbp-1* sequence that do not align to *cbp-2* or *cbp-3* are not shown. b) Protein domains encoded in CBP-1, CBP-2, and CBP-3. Orange represents zinc finger domains, blue represents KIX domains, green represents a bromodomain, and yellow represents a HAT domain. Numbers below indicate amino acid number. c) Fold change of *cbp-1* upon *cbp-1*, *cbp-2*, and *cbp-3* knockdowns compared to vector RNAi. Bars represent the mean of three biological replicates, error bars represent one standard deviation. d) Photos of fourteen strains with vector control, *cbp-1*, *cbp-2*, and *cbp-3* RNAi. Strains that do not interact with *cbp-1* are not shown. Blue border indicates fluorescence decrease and orange border indicates fluorescence increase. Scale bar = 100 µM.

As discussed above, the CBP/p300 ortholog *cbp-1* regulates the greatest number of the tested promoters in the screen. This CF has been extensively studied in many eukaryotic model systems and was found here to function as both a transcriptional activator and repressor (Boija *et al*. 2017; Hunt *et al*. 2022). In early *C. elegans* development, *cbp-1* is required for proper cell fate decisions (Shi and Mello 1998). The *C. elegans* genome also encodes two shorter CBP/p300 homologs, *cbp-2* and *cbp-3.* All three *C. elegans cbp* genes are located within a 160 kb stretch of chromosome III and are likely the result of partial gene duplications, as they share sequence homology (Fig. 6a). Although not annotated in WormBase as such, both *cbp-2* and *cbp-3* have previously been classified as pseudogenes, although both are expressed at the mRNA level in the intestine (Shi and Mello 1998; Mitrovich and Anderson 2005; Cao *et al*. 2017). We examined InterPro annotations for protein domains and found that CBP-1 has four zinc finger domains, a RING domain, a KIX domain, a bromodomain, and a large HAT domain (Fig. 6b). However, CBP-2 only contains the first zinc finger domain and the KIX domain, and CBP-3 only contains the first zinc finger domain (Fig. 6b). According to these annotations, neither CBP-2 nor CBP-3 would have acetyltransferase activity on their own. In our screen, knockdown of all three *cbp* paralogs individually showed an effect on a subset of promoters. However, we noticed that the *cbp-2* clone we obtained from the ORFeome library (Rual *et al*. 2004) actually contained the *cbp-3* sequence. The sequences of all three *cbp* genes closely align, and the 5′ and 3′ ends of *cbp-2* and *cbp-3* are particularly similar (Fig. 6a). We created a new *cbp-2* RNAi clone that targets a more unique sequence within the *cbp-2* mRNA and verified that both this and the RNAi clone for *cbp-3* do not affect *cbp-1* expression (Fig. 6c). We then tested the new *cbp-2* RNAi clone together with the clones for *cbp-1* and *cbp-3* to determine whether all three paralogs interact with the same 14 promoters that were affected by *cbp-1* RNAi (Fig. 6d). Although neither *cbp-2* nor *cbp-3* affected all fourteen reporters, both did regulate multiple promoters. While these results do not unveil the extent to which the *cbp* paralogs act in *C. elegans* genetic regulation, these findings indicate that *cbp-2* and *cbp-3* may produce functional proteins and may not be pseudogenes.

## Discussion

In this study, we generated a comprehensive RNAi resource for *C. elegans* CFs and examined how the depletion of each of these affects promoter activity in the *C. elegans* intestine. There are clear advantages to our promoter-centered approach, including the ability to comprehensively test the effects of regulators such as CFs, the ability to specifically examine changes in one tissue in living animals, and the knowledge of the DNA element through which the effect on reporter expression occurs, in this case usually ∼2 kb gene promoters. However, there are several distinct technical and conceptual disadvantages as well. Technical challenges include the fact that high-throughput RNAi screens can be noisy, i.e. they miss interactions. This is because the screens are done visually, because not all promoter reporters are integrated into the genome, resulting in mosaicism of fluorescence, and because some promoters drive low levels of fluorescent protein expression, which makes it more difficult to observe reductions in fluorescence upon RNAi of a regulator. A notable conceptual disadvantage includes the focus on a single tissue only during development. Additionally, we began RNAi at the L1 stage when many CF-encoding genes may have already produced protein and therefore functional protein could persist during the time of knockdown. Further, we cannot rule out redundancy exhibited by members of the same complex or different CFs that could compensate for the loss of a specific CF. Finally, we acknowledge that our RNAi may not deplete mRNA to levels that cause a phenotype. As an indication of data quality, we were able to recapitulate known regulatory interactions between CFs and promoters, such as *mdt-15* and *cbp-1* knockdowns decreasing *gst-4* levels (Goh *et al*. 2014; Hou *et al*. 2014; Frankino *et al*. 2022), as well as *mdt-15* knockdowns activating *hsp-4* expression (Hou *et al*. 2014).

Of note, many of the promoters used in this study are stress inducible. Therefore, it is possible that the lack of effect seen upon knockdown for some activating CFs may be attributed to the gene not being expressed under basal conditions.

Overall, we observed very few changes with the knockdown of methyltransferases, demethylases, HDACs, remodelers, or TBP-associated proteins. Instead, the majority of expression changes occurred through depletion of HATs, RNA pol-II-associated factors, or Mediator components (Fig. 3). However, because we only used 19 promoters, it remains to be determined how generalizable these observations are. Several studies have revealed that many CFs regulate specific genes in other organisms (Lenstra *et al*. 2011; Haberle *et al*. 2019; Neumayr *et al*. 2022). Since we also find that many CFs act specifically on some promoters without detectable effects on others, these findings together indicate that many CFs act in a highly gene-specific manner.

We observed little coherence in promoters affected by different members of the same CF complex. However, for some CF complexes multiple members did regulate the same promoters (Fig. 4). The two promoters whose activity was changed by depletion of the most CFs were *Pacdh-1* and *Pacdh-2*. Interestingly, these two promoters were also most extensively affected by TF and metabolic enzyme RNAi, indicating that they may be tightly regulated and that they are sensitive to changes in the environment or condition of the animal. The most connected CFs are *cbp-1* and CCR4-NOT complex components. All members of the CCR4-NOT complex affected at least one promoter when knocked down by RNAi, and several affected multiple promoters. Interestingly, knockdown of some CCR4-NOT members affected *Pacdh-2::GFP* expression differently; some increased promoter activity while others elicited a decrease. The CCR4-NOT complex functions at several steps in gene expression and the different effects on the *Pacdh-2* promoter could be due to the different proteins contributing to these differential functions (Collart 2016). In addition, knockdown of several members of both the CCR4-NOT and NuA4 complexes increased *Phsp-4::GFP* activity. This could indicate that these complexes function together to regulate the expression of this stress-responsive promoter. As mentioned above, the CCR4-NOT complex functions in several processes including mRNA deadenylation and degradation, transcriptional initiation and elongation, and mRNA export. The NuA4 HAT complex acetylates both H4 and H2A, leading to transcriptional activation. Certain members of the CCR4-NOT complex are found associated with chromatin at promoters and transcriptional start site-proximal regions and contribute to global acetylation in yeast (Peng *et al*. 2008; Venters *et al*. 2011). Therefore, it is possible that these two complexes function together to regulate acetylation at the *hsp-4* promoter. Alternatively, given the other potential roles of CCR4-NOT, it is possible that these two complexes function independently to regulate *hsp-4* expression.

The CF that regulated the greatest number of promoters was *cbp-1,* which affected 14 of the 19 promoters. While its ortholog p300/CBP has been mostly known for its role in transcriptional activation through its acetyltransferase activity, it has recently been shown to have repressive functions independent of this enzymatic activity as well (Hunt *et al*. 2022). Two paralogs of *cbp-1*, *cbp-2*, and *cbp-3*, were previously annotated as pseudogenes (Shi and Mello 1998; Mitrovich and Anderson 2005). However, we found that independent depletion of either of these genes affected promoter activity. Neither of the proteins encoded by these homologs are predicted to have a HAT domain, indicating that they act by other mechanisms. Future work will be needed to further characterize the biological function of these two genes and how they affect gene expression.

We previously used the same 19 reporter strains to identify TFs and metabolic genes that affect promoter activity (MacNeil *et al*. 2015; Bhattacharya *et al*. 2022). While depletion of CFs or TFs frequently decreased promoter activity, knockdown of metabolic genes generally resulted in increased promoter activity (MacNeil *et al*. 2015; Bhattacharya *et al*. 2022) (this study). In the TF study, we found that many TFs affected promoter activity indirectly, i.e. without apparent physical binding. Since metabolism and gene expression frequently influence each other (Watson *et al*. 2015; Li *et al*. 2018; Giese *et al*. 2019; Carthew 2021), we hypothesized that metabolism may connect TFs that indirectly regulate promoter activity to TFs that both bind and regulate promoter activity. Similarly, we reasoned that because CFs often connect metabolism and gene regulation, for instance by modifying DNA or histones with metabolites such as methyl and acetyl groups (van der Knaap and Verrijzer 2016), we may be able to place these transcriptional regulators in the context of larger GRNs comprising promoters, TFs, metabolic genes, and CFs. Previously, we used epistasis-based nested effects modeling to organize the TF-based GRN into a hierarchy that depicts the regulatory ‘flow of information’ (Markowetz *et al*. 2007; MacNeil *et al*. 2015). Here, we were unable to use the same approach to connect TFs with CFs and metabolic genes. This could be because of the size of the datasets that need to be combined, because the data are relatively noisy, and/or because the output of nested effects modeling is difficult to navigate. We propose that it may be more feasible to build regulatory networks for individual promoters, one-at-a-time, to incorporate different types of regulators.

We explored the concept of combining different types of regulators for individual promoters with the *acdh-1* promoter, which is affected by the greatest number of TFs, CFs, and metabolic genes and which we have previously studied in more detail (Watson *et al*. 2013; Watson *et al*. 2014; Bulcha *et al*. 2019; Giese *et al*. 2020; Bhattacharya *et al*. 2022) (this study). There are currently at least three known mechanisms of *acdh-1* activation, some of which we have studied in detail, and some of which remain to be elucidated further. First, *acdh-1*, like all 19 promoters, is activated by the intestinal master regulator *elt-2*. This GATA TF activates genes, including those encoding TFs, and resides at the top of the regulatory hierarchy (MacNeil *et al*. 2015). The SREBP ortholog, *sbp-1*, induces additional TFs and also activates *acdh-1*. These TFs include *nhr-68*, which acts in a type I coherent feed-forward loop with *nhr-10* to activate *acdh-1* in response to sustained propionate accumulation (Ding *et al*. 2015; Bulcha *et al*. 2019). SBP-1 also activates *nhr-114*, which activates *acdh-1* in response to low Met/SAM cycle flux (Giese *et al*. 2020). Here, we found that different CFs do not appear to provide additional specificity to *acdh-1* activation in response to different metabolic perturbations. Therefore, the molecular mechanisms by which TFs and CFs converge on the *acdh-1* promoter under different metabolic perturbations, and how these regulatory effects are insulated from each other remain to be elucidated, for instance by using chromatin-immunoprecipitation-based methods. Finally, using more genome-scale methods such as perturb-seq may shed further light on the interplay between TFs, CFs, and metabolic genes.

## Supplementary Material

jkad096_Supplementary_Data

## Data Availability

All interaction data generated in this study are available in the article and associated supplementary files. The CF RNAi library is available from the corresponding author upon request. Supplemental material available at G3 online.

## References

[jkad096-B1] Adamson B , Norman TM, Jost M, Cho MY, Nunez JK, Chen Y, Villalta JE, Gilbert LA., Horlbeck MA., Hein MY, et al A multiplexed single-cell CRISPR screening platform enables systematic dissection of the unfolded protein response. Cell. 2016;167(7):1867–1882.e21. doi:10.1016/j.cell.2016.11.048. 27984733 PMC5315571

[jkad096-B2] Anandhakumar J , Moustafa YW, Chowdhary S, Kainth AS, Gross DS. Evidence for multiple mediator complexes in yeast independently recruited by activated heat shock factor. Mol Cell Biol. 2016;36(14):1943–1960. doi:10.1128/MCB.00005-16. 27185874 PMC4936062

[jkad096-B3] Araya CL , Kawli T, Kundaje A, Jiang L, Wu B, Vafeados D, Terrell R, Weissdepp P, Gevirtzman L, Mace D, et al Regulatory analysis of the *C. elegans* genome with spatiotemporal resolution. Nature. 2014;512(7515):400–405. doi:10.1038/nature13497. 25164749 PMC4530805

[jkad096-B4] Arda HE , Taubert S, MacNeil LT, Conine CC, Tsuda B, Van Gilst MR, Sequerra R, Doucette-Stamm L, Yamamoto KR, Walhout AJ. Functional modularity of nuclear hormone receptors in a *C. elegans* gene regulatory network. Mol Syst Biol. 2010;6(1):367. doi:10.1038/msb.2010.23. 20461074 PMC2890327

[jkad096-B5] Ashburner M , Ball CA, Blake JA, Botstein D, Butler H, Cherry JM, Davis AP, Dolinski K, Dwight SS, Eppig JT, et al Gene ontology: tool for the unification of biology. The Gene Ontology Consortium. Nat Genet. 2000;25(1):25–29. doi:10.1038/75556. 10802651 PMC3037419

[jkad096-B6] Bannister AJ , Kouzarides T. Regulation of chromatin by histone modifications. Cell Res. 2011;21(3):381–395. doi:10.1038/cr.2011.22. 21321607 PMC3193420

[jkad096-B7] Bhattacharya S , Horowitz BB, Zhang J, Li X, Zhang H, Giese GE, Holdorf AD, Walhout AJ. A metabolic regulatory network for the *Caenorhabditis elegans* intestine. iScience. 2022;25(8):104688. doi:10.1016/j.isci.2022.104688. PMC9283940 35847555

[jkad096-B8] Blum M , Chang HY, Chuguransky S, Grego T, Kandasaamy S, Mitchell A, Nuka G, Paysan-Lafosse T, Qureshi M, Raj S, et al The InterPro protein families and domains database: 20 years on. Nucleic Acids Res. 2021;49(D1):D344–D354. doi:10.1093/nar/gkaa977. 33156333 PMC7778928

[jkad096-B9] Boija A , Mahat DB, Zare A, Holmqvist PH, Philip P, Meyers DJ, Cole PA, Lis JT, Stenberg P, Mannervik M. CBP Regulates recruitment and release of promoter-proximal RNA polymerase II. Mol Cell. 2017;68(3):491–503.e5. doi:10.1016/j.molcel.2017.09.031. 29056321 PMC5826544

[jkad096-B10] Bourbon HM . Comparative genomics supports a deep evolutionary origin for the large, four-module transcriptional mediator complex. Nucleic Acids Res. 2008;36(12):3993–4008. doi:10.1093/nar/gkn349. 18515835 PMC2475620

[jkad096-B11] Brejc K , Bian Q, Uzawa S, Wheeler BS, Anderson EC, King DS, Kranzusch PJ, Preston CG, Meyer BJ. Dynamic control of X chromosome conformation and repression by a histone H4K20 demethylase. Cell. 2017;171(1):85–102.e23. doi:10.1016/j.cell.2017.07.041. 28867287 PMC5678999

[jkad096-B12] Brenner S . The genetics of *Caenorhabditis elegans*. Genetics. 1974;77(1):71–94. doi:10.1093/genetics/77.1.71. 4366476 PMC1213120

[jkad096-B13] Bulcha JT , Giese GE, Ali MZ, Lee Y-U, Walker MD, Holdorf AD, Yilmaz LS, Brewster RC, Walhout AJ. A persistence detector for metabolic network rewiring in an animal. Cell Rep. 2019;26(2):460–468. doi:10.1016/j.celrep.2018.12.064. 30625328 PMC6368391

[jkad096-B14] Cai G , Imasaki T, Takagi Y, Asturias FJ. Mediator structural conservation and implications for the regulation mechanism. Structure. 2009;17(4):559–567. doi:10.1016/j.str.2009.01.016. 19368889 PMC2673807

[jkad096-B15] Cao J , Packer JS, Ramani V, Cusanovich DA, Huynh C, Daza R, Qiu X, Lee C, Furlan SN, Steemers FJ, et al Comprehensive single-cell transcriptional profiling of a multicellular organism. Science. 2017;357(6352):661–667. doi:10.1126/science.aam8940. 28818938 PMC5894354

[jkad096-B16] Carthew RW . Gene regulation and cellular metabolism: an essential partnership. Trends Genet. 2021;37(4):389–400. doi:10.1016/j.tig.2020.09.018. 33092903 PMC7969386

[jkad096-B17] Chalfie M , Tu Y, Euskirchen G, Ward WW, Prasher DC. Green fluorescent protein as a marker for gene expression. Science. 1994;263(5148):802–805. doi:10.1126/science.8303295. 8303295

[jkad096-B18] Collart MA . The Ccr4-Not complex is a key regulator of eukaryotic gene expression. Wiley Interdiscip Rev RNA. 2016;7(4):438–454. doi:10.1002/wrna.1332. 26821858 PMC5066686

[jkad096-B19] Conte D Jr , MacNeil LT, Walhout AJ, Mello CC. RNA Interference in *Caenorhabditis elegans*. Curr Protoc Mol Biol. 2015;109(1):26.23.21–26.23.30. doi:10.1002/0471142727.mb2603s109. PMC5396541 25559107

[jkad096-B20] Cramer P . Organization and regulation of gene transcription. Nature. 2019;573(7772):45–54. doi:10.1038/s41586-019-1517-4. 31462772

[jkad096-B21] Cui M , Han M. Roles of chromatin factors in *C. elegans* development. Wormbook. 2007:1–16. doi:10.1895/wormbook.1.139.1. PMC4781364 18050494

[jkad096-B22] Ding W , Smulan LJ, Hou NS, Taubert S, Watts JL, Walker AK. s-Adenosylmethionine levels govern innate immunity through distinct methylation-dependent pathways. Cell Metab. 2015;22(4):633–645. doi:10.1016/j.cmet.2015.07.013. 26321661 PMC4598287

[jkad096-B23] Dixit A , Parnas O, Li B, Chen J, Fulco CP, Jerby-Arnon L, Marjanovic ND, Dionne D, Burks T, Raychowdhury R, et al Perturb-Seq: dissecting molecular circuits with scalable single-cell RNA profiling of pooled genetic screens. Cell. 2016;167(7):1853–1866.e17. doi:10.1016/j.cell.2016.11.038. 27984732 PMC5181115

[jkad096-B24] El Khattabi L , Zhao H, Kalchschmidt J, Young N, Jung S, Van Blerkom P, Kieffer-Kwon P, Kieffer-Kwon K-R, Park S, Wang X, et al A pliable mediator acts as a functional rather than an architectural bridge between promoters and enhancers. Cell. 2019;178(5):1145–1158.e20. doi:10.1016/j.cell.2019.07.011. 31402173 PMC7533040

[jkad096-B25] Frankino PA , Siddiqi TF, Bolas T, Bar-Ziv R, Gildea HK, Zhang H, Higuchi-Sanabria R, Dillin A. SKN-1 regulates stress resistance downstream of amino catabolism pathways. iScience. 2022;25(7):104571. doi:10.1016/j.isci.2022.104571. PMC9240870 35784796

[jkad096-B26] Fraser AG , Kamath RS, Zipperlen P, Martinez-Campos M, Sohrmann M, Ahringer J. Functional genomics analysis of *C. elegans* chromosome I by systematic RNA interference. Nature. 2000;408(6810):325–330. doi:10.1038/35042517. 11099033

[jkad096-B27] Fuxman Bass JI , Pons C, Kozlowski L, Reece-Hoyes JS, Shrestha S, Holdorf AD, Mori A, Myers CL, Walhout AJ. A gene-centered *C. elegans* protein-DNA interaction network provides a framework for functional predictions. Mol Syst Biol. 2016;12(10):884. doi:10.15252/msb.20167131. 27777270 PMC5081483

[jkad096-B28] Fuxman Bass JI , Sahni N, Shrestha S, Garcia-Gonzalez A, Mori A, Bhat N, Yi S, Hill DE, Vidal M, Walhout AJ. Human gene-centered transcription factor networks for enhancers and disease variants. Cell. 2015;161(3):661–673. doi:10.1016/j.cell.2015.03.003. 25910213 PMC4409666

[jkad096-B29] Giese GE , Nanda S, Holdorf AD, Walhout AJ. Transcriptional regulation of metabolic flux: a *C. elegans* perspective. Curr Opin Syst Biol. 2019;15:12–18. doi:10.1016/j.coisb.2019.03.002.

[jkad096-B30] Giese GE , Walker MD, Ponomarova O, Zhang H, Li X, Minevich G, Walhout AJ. *Caenorhabditis elegans* methionine/S-adenosylmethionine cycle activity is sensed and adjusted by a nuclear hormone receptor. Elife. 2020;9:e60259. doi:10.7554/eLife.60259. PMC7561351 33016879

[jkad096-B31] Goh GY , Martelli KL, Parhar KS, Kwong AW, Wong MA, Mah A, Hou NS, Taubert S. The conserved mediator subunit MDT-15 is required for oxidative stress responses in *Caenorhabditis elegans*. Aging Cell. 2014;13(1):70–79. doi:10.1111/acel.12154. 23957350 PMC4326869

[jkad096-B32] Haberle V , Arnold CD, Pagani M, Rath M, Schernhuber K, Stark A. Transcriptional cofactors display specificity for distinct types of core promoters. Nature. 2019;570(7759):122–126. doi:10.1038/s41586-019-1210-7. 31092928 PMC7613045

[jkad096-B33] Haberle V , Stark A. Eukaryotic core promoters and the functional basis of transcription initiation. Nat Rev Mol Cell Biol. 2018;19(10):621–637. doi:10.1038/s41580-018-0028-8. 29946135 PMC6205604

[jkad096-B34] Halter D , Collart MA, Panasenko OO. The Not4 E3 ligase and CCR4 deadenylase play distinct roles in protein quality control. PLoS One. 2014;9(1):e86218. doi:10.1371/journal.pone.0086218. PMC3895043 24465968

[jkad096-B35] Hendy O , Serebreni L, Bergauer K, Muerdter F, Huber L, Nemčko F, Stark A. Developmental and housekeeping transcriptional programs in Drosophila require distinct chromatin remodelers. Mol Cell. 2022;82(19):3598–3612.e7. doi:10.1016/j.molcel.2022.08.019. 36113480 PMC7614073

[jkad096-B36] Hou NS , Gutschmidt A, Choi DY, Pather K, Shi X, Watts JL, Hoppe T, Taubert S. Activation of the endoplasmic reticulum unfolded protein response by lipid disequilibrium without disturbed proteostasis in vivo. Proc Natl Acad Sci U S A. 2014;111(22):E2271–E2280. doi:10.1073/pnas.1318262111. 24843123 PMC4050548

[jkad096-B37] Hunt G , Boija A, Mannervik M. P300/CBP sustains polycomb silencing by non-enzymatic functions. Mol Cell. 2022;82(19):3580–3597.e9. doi:10.1016/j.molcel.2022.09.005. 36206738

[jkad096-B38] Johnson DS , Mortavi A, Myers RM, Wold B. Genome-wide mapping of *in vivo* protein-DNA interactions. Science. 2007;316(5830):1497–1502. doi:10.1126/science.1141319. 17540862

[jkad096-B39] Kaletsky R , Yao V, Williams A, Runnels AM, Tadych A, Zhou S, Troyanskaya OG, Murphy CT. Transcriptome analysis of adult *Caenorhabditis elegans* cells reveals tissue-specific gene and isoform expression. PLoS Genet. 2018;14(8):e1007559. doi:10.1371/journal.pgen.1007559. PMC6105014 30096138

[jkad096-B40] Kamath RS , Fraser AG, Dong Y, Poulin G, Durbin R, Gotta M, Kanapin A, Le Bot N, Moreno S, Sohrmann M, et al Systematic functional analysis of the *Caenorhabditis elegans* genome using RNAi. Nature. 2003;421(6920):231–237. doi:10.1038/nature01278. 12529635

[jkad096-B41] Kemmeren P , Sameith K, van de Pasch LA, Benschop JJ, Lenstra TL, Margaritis T, O’Duibhir E, Apweiler E, van Wageningen S, Ko CW, et al Large-scale genetic perturbations reveal regulatory networks and an abundance of gene-specific repressors. Cell. 2014;157(3):740–752. doi:10.1016/j.cell.2014.02.054. 24766815

[jkad096-B42] Lee RYN , Howe KL, Harris TW, Arnaboldi V, Cain S, Chan J, Chen WJ, Davis P, Gao S, Grove C, et al Wormbase 2017: molting into a new stage. Nucleic Acids Res. 2018;46(D1):D869–D874. doi:10.1093/nar/gkx998. 29069413 PMC5753391

[jkad096-B43] Lehner B , Crombie C, Tischler J, Fortunato A, Fraser AG. Systematic mapping of genetic interactions in *Caenorhabditis elegans* identifies common modifiers of diverse signaling pathways. Nat Genet. 2006;38(8):896–903. doi:10.1038/ng1844. 16845399

[jkad096-B44] Lenstra TL , Benschop JJ, Kim T, Schulze JM, Brabers NA, Margaritis T, van de Pasch LA, van Heesch SA, Brok MO, Groot Koerkamp MJ, et al The specificity and topology of chromatin interaction pathways in yeast. Mol Cell. 2011;42(4):536–549. doi:10.1016/j.molcel.2011.03.026. 21596317 PMC4435841

[jkad096-B45] Li X , Egervari G, Wang Y, Berger SL, Lu Z. Regulation of chromatin and gene expression by metabolic enzymes and metabolites. Nat Rev Mol Cell Biol. 2018;19(9):563–578. doi:10.1038/s41580-018-0029-7. 29930302 PMC6907087

[jkad096-B46] Li X , Seidel CW, Szerszen LT, Lange JJ, Workman JL, Abmayr SM. Enzymatic modules of the SAGA chromatin-modifying complex play distinct roles in Drosophila gene expression and development. Genes Dev. 2017;31(15):1588–1600. doi:10.1101/gad.300988.117. 28887412 PMC5630023

[jkad096-B47] Liu B , Jing Z, Zhang X, Chen Y, Mao S, Kaundal R, Zou Y, Wei G, Zang Y, Wang X, et al Large-scale multiplexed mosaic CRISPR perturbation in the whole organism. Cell. 2022;185(16):3008–3024.e16. doi:10.1016/j.cell.2022.06.039. 35870449

[jkad096-B48] Livak KJ , Schmittgen TD. Analysis of relative gene expression data using real-time quantitative PCR and the 2(-Delta Delta C(T)) method. Methods. 2001;25(4):402–408. doi:10.1006/meth.2001.1262. 11846609

[jkad096-B49] Lu PY , Levesque N, Kobor MS. Nua4 and SWR1-C: two chromatin-modifying complexes with overlapping functions and components. Biochem Cell Biol. 2009;87(5):799–815. doi:10.1139/O09-062. 19898529

[jkad096-B50] MacNeil LT , Pons C, Arda HE, Giese GE, Myers CL, Walhout AJ. Transcription factor activity mapping of a tissue-specific gene regulatory network. Cell Syst. 2015;1(2):152–162. doi:10.1016/j.cels.2015.08.003. 26430702 PMC4584425

[jkad096-B51] MacNeil LT , Watson E, Arda HE, Zhu LJ, Walhout AJ. Diet-induced developmental acceleration independent of TOR and insulin in *C. elegans*. Cell. 2013;153(1):240–252. doi:10.1016/j.cell.2013.02.049. 23540701 PMC3821073

[jkad096-B52] Markowetz F , Kostka D, Troyanskaya OG, Spang R. Nested effects models for high-dimensional phenotyping screens. Bioinformatics. 2007;23(13):i305–i312. doi:10.1093/bioinformatics/btm178. 17646311

[jkad096-B53] McIsaac RS , Petti AA, Bussemaker HJ, Botstein D. Perturbation-based analysis and modeling of combinatorial regulation in the yeast sulfur assimilation pathway. Mol Biol Cell. 2012;23(15):2993–3007. doi:10.1091/mbc.e12-03-0232. 22696683 PMC3408425

[jkad096-B54] Mitrovich QM , Anderson P. mRNA surveillance of expressed pseudogenes in *C. elegans*. Curr Biol. 2005;15(10):963–967. doi:10.1016/j.cub.2005.04.055. 15916954

[jkad096-B55] Neumayr C , Haberle V, Serebreni L, Karner K, Hendy O, Boija A, Henninger JE, Li CH, Stejskal K, Lin G, et al Differential cofactor dependencies define distinct types of human enhancers. Nature. 2022;606(7913):406–413. doi:10.1038/s41586-022-04779-x. 35650434 PMC7613064

[jkad096-B56] Peng W , Togawa C, Zhang K, Kurdistani SK. Regulators of cellular levels of histone acetylation in Saccharomyces cerevisiae. Genetics. 2008;179(1):277–289. doi:10.1534/genetics.107.085068. 18493053 PMC2390606

[jkad096-B57] Reece-Hoyes JS , Deplancke B, Shingles J, Grove CA, Hope IA, Walhout AJ. A compendium of *C. elegans* regulatory transcription factors: a resource for mapping transcription regulatory networks. Genome Biol. 2005;6(13):R110. doi:10.1186/gb-2005-6-13-r110. PMC1414109 16420670

[jkad096-B58] Reece-Hoyes JS , Pons C, Diallo A, Mori A, Shrestha S, Kadreppa S, Nelson J, DiPrima S, Dricot A, Lajoie BR, et al Extensive rewiring and complex evolutionary dynamics in a *C. elegans* multiparameter transcription factor network. Mol Cell. 2013;51(1):116–127. doi:10.1016/j.molcel.2013.05.018. 23791784 PMC3794439

[jkad096-B59] Rogers JM , Bulyk ML. Diversification of transcription factor-DNA interactions and the evolution of gene regulatory networks. Wiley Interdiscip Rev Syst Biol Med:. 2018;10(5):e1423. doi:10.1002/wsbm.1423. PMC6202284 29694718

[jkad096-B60] Rual J-F , Ceron J, Koreth J, Hao T, Nicot A-S, Hirozane-Kishikawa T, Vandenhaute J, Orkin SH, Hill DE, van den Heuvel S, et al Toward improving *Caenorhabditis elegans* phenome mapping with an ORFeome-based RNAi library. Genome Res. 2004;14(10b):2162–2168. doi:10.1101/gr.2505604. 15489339 PMC528933

[jkad096-B61] Shi Y , Mello C. A CBP/p300 homolog specifies multiple differentiation pathways in *Caenorhabditis elegans*. Genes Dev. 1998;12(7):943–955. doi:10.1101/gad.12.7.943. 9531533 PMC316678

[jkad096-B62] Sonawane AR , Platig J, Fagny M, Chen CY, Paulson JN, Lopes-Ramos CM, DeMeo DL, Quackenbush J, Glass K, Kuijjer ML, et al Understanding tissue-specific gene regulation. Cell Rep. 2017;21(4):1077–1088. doi:10.1016/j.celrep.2017.10.001. 29069589 PMC5828531

[jkad096-B63] Subramaniam N , Treuter E, Okret S. Receptor interacting protein RIP140 inhibits both positive and negative gene regulation by glucocorticoids. J Biol Chem. 1999;274(25):18121–18127. doi:10.1074/jbc.274.25.18121. 10364267

[jkad096-B64] Talbert PB , Meers MP, Henikoff S. Old cogs, new tricks: the evolution of gene expression in a chromatin context. Nat Rev Genet. 2019;20(5):283–297. doi:10.1038/s41576-019-0105-7. 30886348

[jkad096-B65] Taubert S , Hansen M, Van Gilst MR, Cooper SB, Yamamoto KR. The mediator subunit MDT-15 confers metabolic adaptation to ingested material. PLoS Genet. 2008;4(2):e1000021. doi:10.1371/journal.pgen.1000021. PMC2265483 18454197

[jkad096-B66] Taubert S , Van Gilst MR, Hansen M, Yamamoto KR. A mediator subunit, MDT-15, integrates regulation of fatty acid metabolism by NHR-49-dependent and -independent pathways in *C. elegans*. Genes Dev. 2006;20(9):1137–1149. doi:10.1101/gad.1395406. 16651656 PMC1472473

[jkad096-B67] Tursun B , Patel T, Kratsios P, Hobert O. Direct conversion of *C. elegans* germ cells into specific neuron types. Science. 2011;331(6015):304–308. doi:10.1126/science.1199082. 21148348 PMC3250927

[jkad096-B68] van der Knaap JA , Verrijzer CP. Undercover: gene control by metabolites and metabolic enzymes. Genes Dev. 2016;30(21):2345–2369. doi:10.1101/gad.289140.116. 27881599 PMC5131776

[jkad096-B69] Venters BJ , Wachi S, Mavrich TN, Andersen BE, Jena P, Sinnamon AJ, Jain P, Rolleri NS, Jiang C, Hemeryck-Walsh C, et al A comprehensive genomic binding map of gene and chromatin regulatory proteins in Saccharomyces. Mol Cell. 2011;41(4):480–492. doi:10.1016/j.molcel.2011.01.015. 21329885 PMC3057419

[jkad096-B70] Villanueva CJ , Waki H, Godio C, Nielsen R, Chou WL, Vargas L, Wroblewski K, Schmedt C, Chao LC, Boyadjian R, et al TLE3 Is a dual-function transcriptional coregulator of adipogenesis. Cell Metab. 2011;13(4):413–427. doi:10.1016/j.cmet.2011.02.014. 21459326 PMC3089971

[jkad096-B71] Walhout AJ . Unraveling transcription regulatory networks by protein-DNA and protein-protein interaction mapping. Genome Res. 2006;16(12):1445–1454. doi:10.1101/gr.5321506. 17053092

[jkad096-B72] Walhout AJ , Temple GF, Brasch MA, Hartley JL, Lorson MA, van den Heuvel S, Vidal M. GATEWAY Recombinational cloning: application to the cloning of large numbers of open reading frames or ORFeomes. Meth Enzymol. 2000;328:575–592. doi:10.1016/S0076-6879(00)28419-X. 11075367

[jkad096-B73] Watson E , MacNeil LT, Arda HE, Zhu LJ, Walhout AJ. Integration of metabolic and gene regulatory networks modulates the *C. elegans* dietary response. Cell. 2013;153(1):253–266. doi:10.1016/j.cell.2013.02.050. 23540702 PMC3817025

[jkad096-B74] Watson E , MacNeil LT, Ritter AD, Yilmaz LS, Rosebrock AP, Caudy AA, Walhout AJ. Interspecies systems biology uncovers metabolites affecting *C. elegans* gene expression and life history traits. Cell. 2014;156(4):759–770. doi:10.1016/j.cell.2014.01.047. 24529378 PMC4169190

[jkad096-B75] Watson E , Olin-Sandoval V, Hoy MJ, Li C-H, Louisse T, Yao V, Mori A, Holdorf AD, Troyanskaya OG, Ralser M, et al Metabolic network rewiring of propionate flux compensates vitamin B12 deficiency in *C. elegans*. Elife. 2016;5:e17670. doi:10.7554/eLife.17670. PMC4951191 27383050

[jkad096-B76] Watson E , Yilmaz LS, Walhout AJ. Understanding metabolic regulation at a systems level: metabolite sensing, mathematical predictions and model organisms. Annu Rev Genet. 2015;49(1):553–575. doi:10.1146/annurev-genet-112414-055257. 26631516

[jkad096-B77] Weiner A , Chen HV, Liu CL, Rahat A, Klien A, Soares L, Gudipati M, Pfeffner J, Regev A, Buratowski S, et al Systematic dissection of roles for chromatin regulators in a yeast stress response. PLoS Biol. 2012;10(7):e1001369. doi:10.1371/journal.pbio.1001369. PMC3416867 22912562

[jkad096-B78] Yang F , Vought BW, Satterlee JS, Walker AK, Jim Sun ZY, Watts JL, DeBeaumont R, Mako Saito R, Hyberts SG, Yang S, et al An ARC/mediator subunit required for SREBP control of cholesterol and lipid homeostasis. Nature. 2006;442(7103):700–704. doi:10.1038/nature04942. 16799563

[jkad096-B79] Yilmaz LS , Li X, Nanda S, Fox B, Schroeder F, Walhout AJ, et al Modeling tissue-relevant *Caenorhabditis elegans* metabolism at network, pathway, reaction, and metabolite levels. Mol Syst Biol. 2020;16(10):e9649. doi:10.15252/msb.20209649. PMC7537831 33022146

