# Supplementary material for: A transcriptional cofactor regulatory network for the C. elegans intestine
Source: G3 (Bethesda). 2023 Apr 29;13(7):jkad096. doi: 10.1093/g3journal/jkad096 (PMC10320766; doi:10.1093/g3journal/jkad096)
Supplement: jkad096_Supplementary_Data [file jkad096_supplementary_data.zip › File_S1_G3-2023-404230.pdf]

Supplemental Table S1. CFs encoded in the *C. elegans* genome.

Supplemental Table S2. Hypergeometric enrichments for essentiality among CF classes, CF binding domain-containing proteins, and for CFs, TFs, and metabolic genes.

Supplemental Table S3. Description of *C. elegans* strains used in the primary screen.

Supplemental Table S4. Interactions between CF RNAi strains and the 19 promoter reporters.

Supplemental Table S5. Hypergeometric enrichments for interactions between CF classes or complexes and promoter reporters.

Supplemental Table S6. Oligonucleotides used in this study.

Pacdh-1::GFP

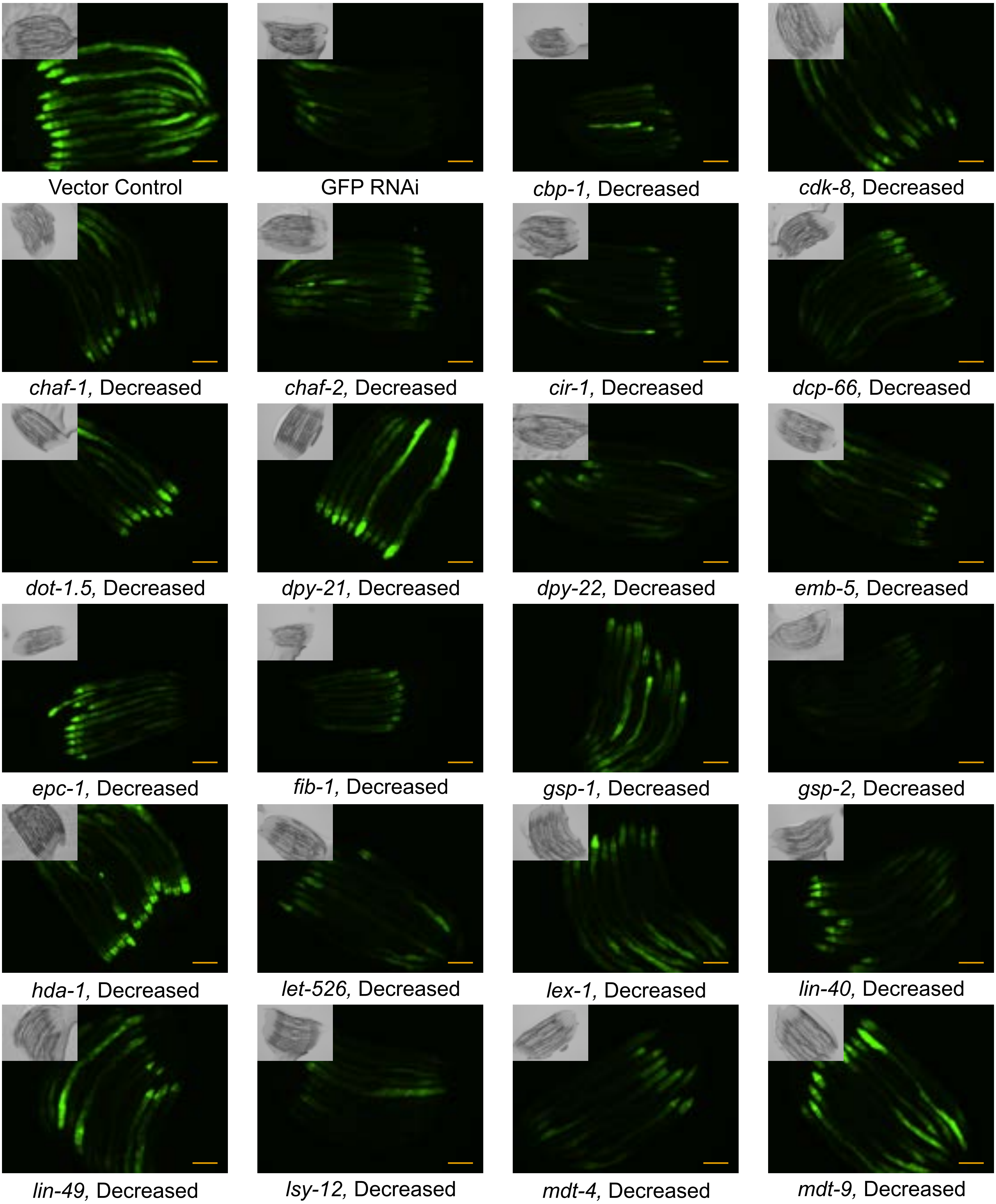
Vector Control
GFP RNAi
cbp-1, Decreased
cdk-8, Decreased
chaf-1, Decreased
chaf-2, Decreased
cir-1, Decreased
dcp-66, Decreased
dot-1.5, Decreased
dpy-21, Decreased
dpy-22, Decreased
emb-5, Decreased
epc-1, Decreased
fib-1, Decreased
gsp-1, Decreased
gsp-2, Decreased
hda-1, Decreased
let-526, Decreased
lex-1, Decreased
lin-40, Decreased
lin-49, Decreased
lsy-12, Decreased
mdt-4, Decreased
mdt-9, Decreased

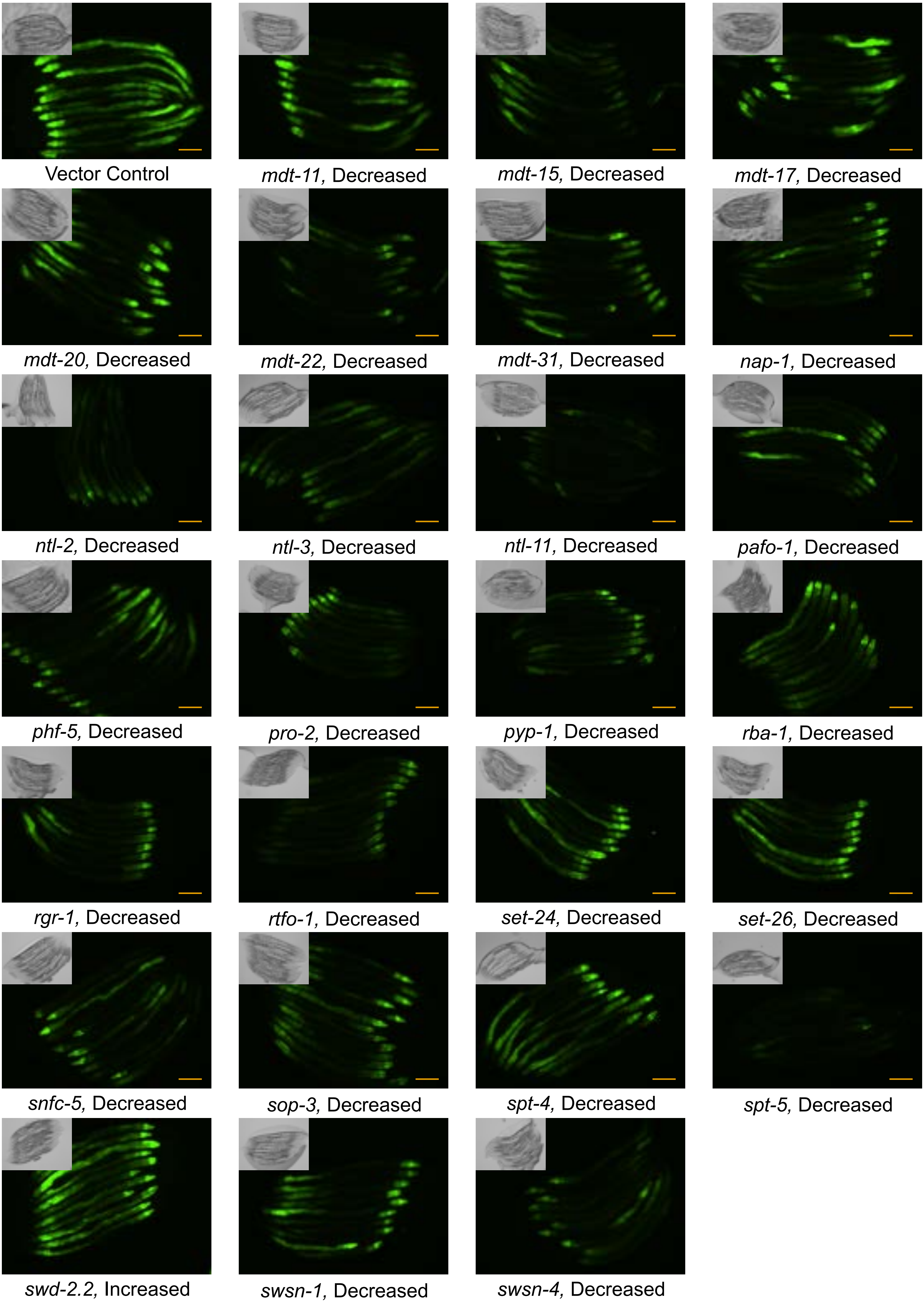
Vector Control
mdt-11, Decreased
mdt-15, Decreased
mdt-17, Decreased
mdt-20, Decreased
mdt-22, Decreased
mdt-31, Decreased
nap-1, Decreased
ntl-2, Decreased
ntl-3, Decreased
ntl-11, Decreased
pafo-1, Decreased
phf-5, Decreased
pro-2, Decreased
pyp-1, Decreased
rba-1, Decreased
rgr-1, Decreased
rtfo-1, Decreased
set-24, Decreased
set-26, Decreased
snfc-5, Decreased
sop-3, Decreased
spt-4, Decreased
spt-5, Decreased
swd-2.2, Increased
swsn-1, Decreased
swsn-4, Decreased

Supplemental Figure S1. RNAi interactions between CFs and *Pacdh-1::GFP.* Scale bar=100μM.

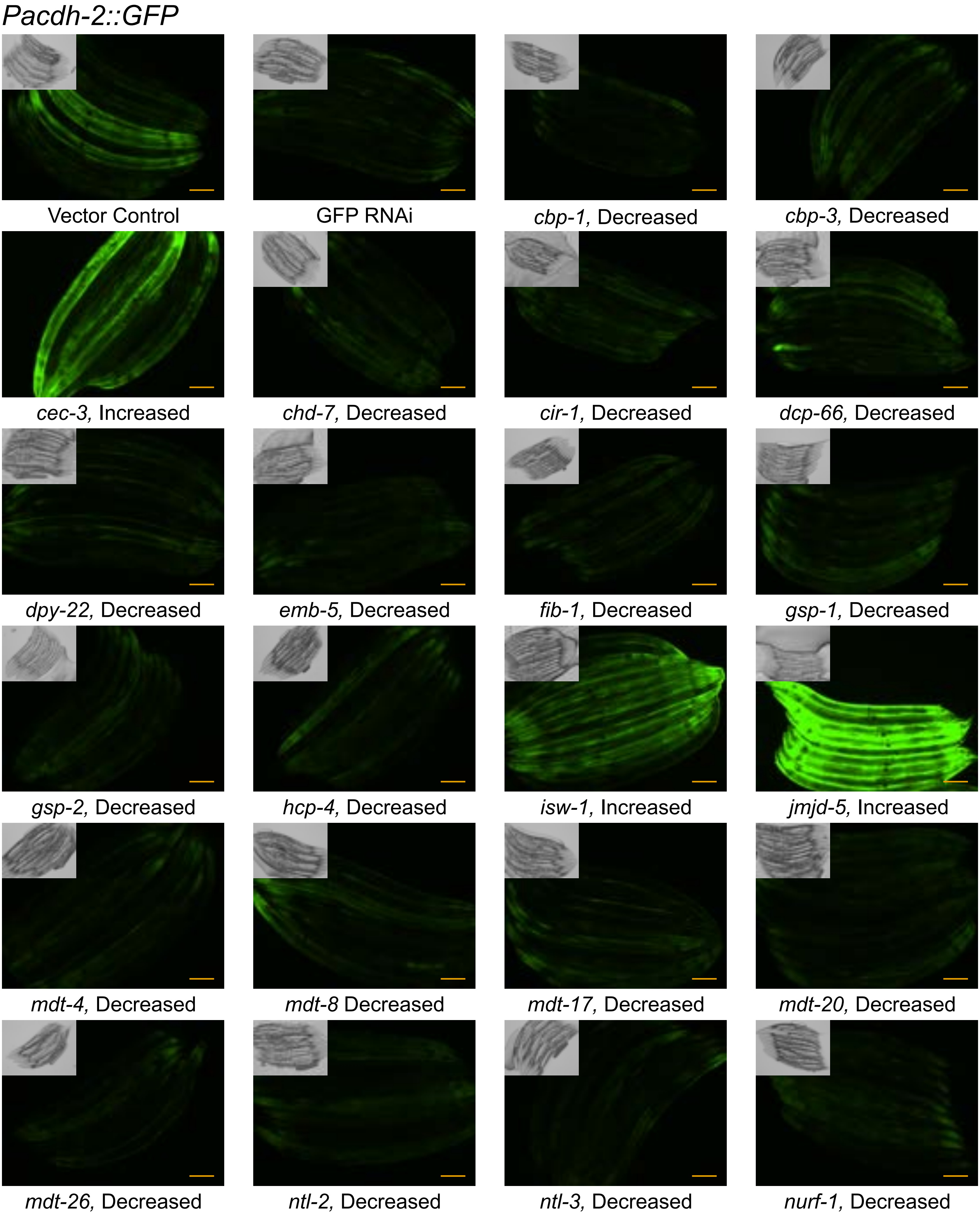
Pacdh-2::GFP
Vector Control
GFP RNAi
cbp-1, Decreased
cbp-3, Decreased
cec-3, Increased
chd-7, Decreased
cir-1, Decreased
dcp-66, Decreased
dpy-22, Decreased
emb-5, Decreased
fib-1, Decreased
gsp-1, Decreased
gsp-2, Decreased
hcp-4, Decreased
isw-1, Increased
jmjd-5, Increased
mdt-4, Decreased
mdt-8 Decreased
mdt-17, Decreased
mdt-20, Decreased
mdt-26, Decreased
ntl-2, Decreased
ntl-3, Decreased
nurf-1, Decreased

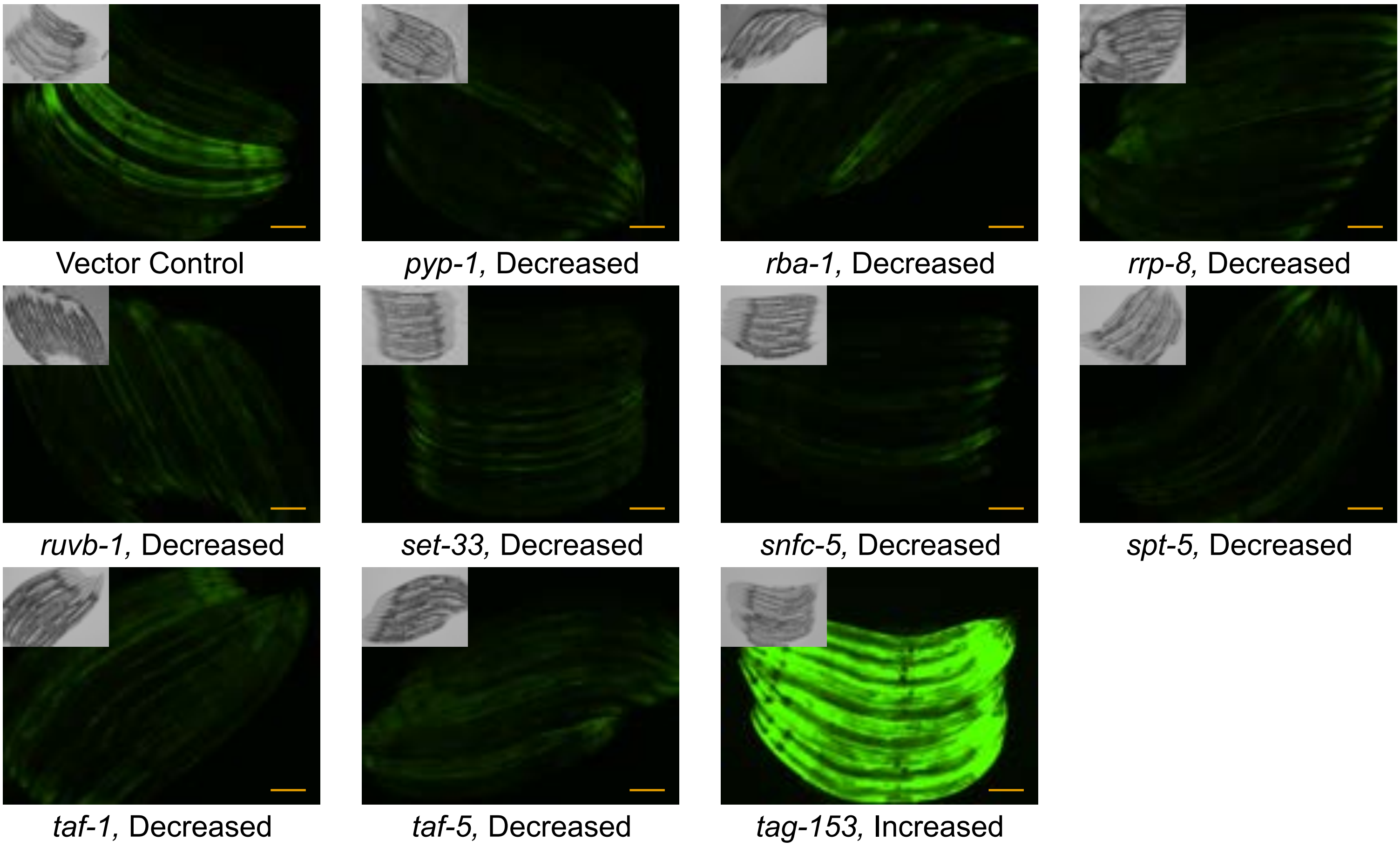
Vector Control
pyp-1, Decreased
rba-1, Decreased
rrp-8, Decreased
ruvb-1, Decreased
set-33, Decreased
snfc-5, Decreased
spt-5, Decreased
taf-1, Decreased
taf-5, Decreased
tag-153, Increased

Supplemental Figure S2. RNAi interactions between CFs and *Pacdh-2::GFP.* Scale bar=100μM.

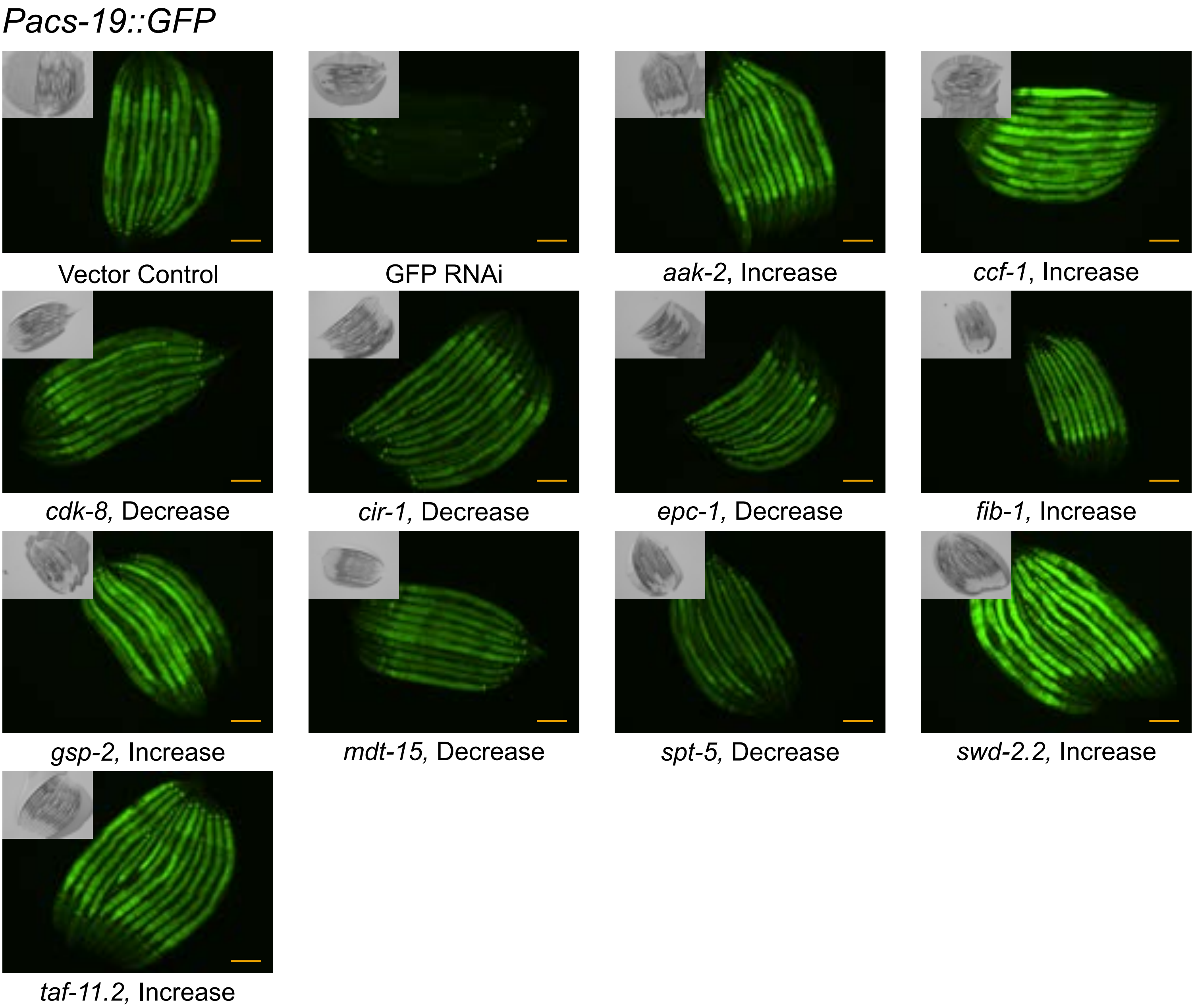

Pacs-19::GFP
Vector Control
GFP RNAi
aak-2, Increase
ccf-1, Increase
cdk-8, Decrease
cir-1, Decrease
epc-1, Decrease
fib-1, Increase
gsp-2, Increase
mdt-15, Decrease
spt-5, Decrease
swd-2.2, Increase
taf-11.2, Increase

Supplemental Figure S3. RNAi interactions between CFs and *Pacs-19::GFP.* Scale bar=100μM.

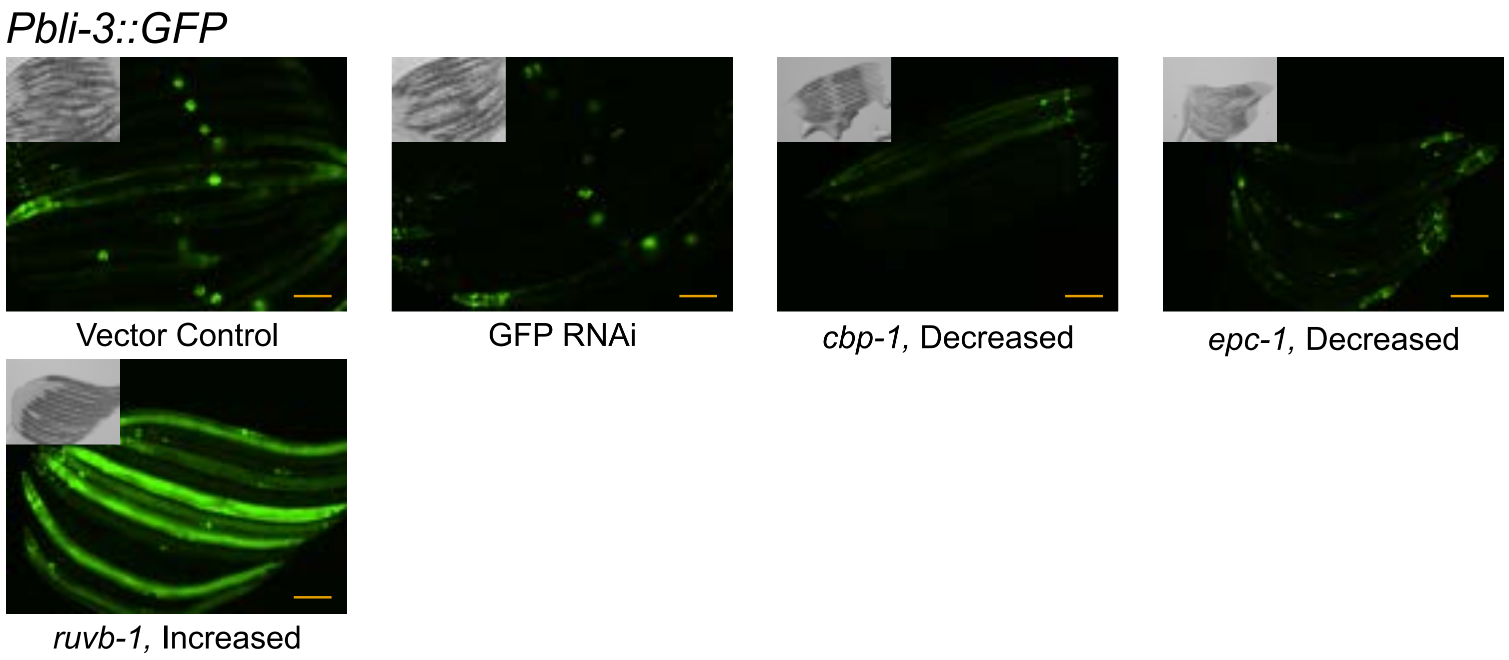
Pbli-3::GFP
Vector Control
GFP RNAi
cbp-1, Decreased
epc-1, Decreased
ruvb-1, Increased

Supplemental Figure S4. RNAi interactions between CFs and *Pbli-3::GFP.* Scale bar=100μM.

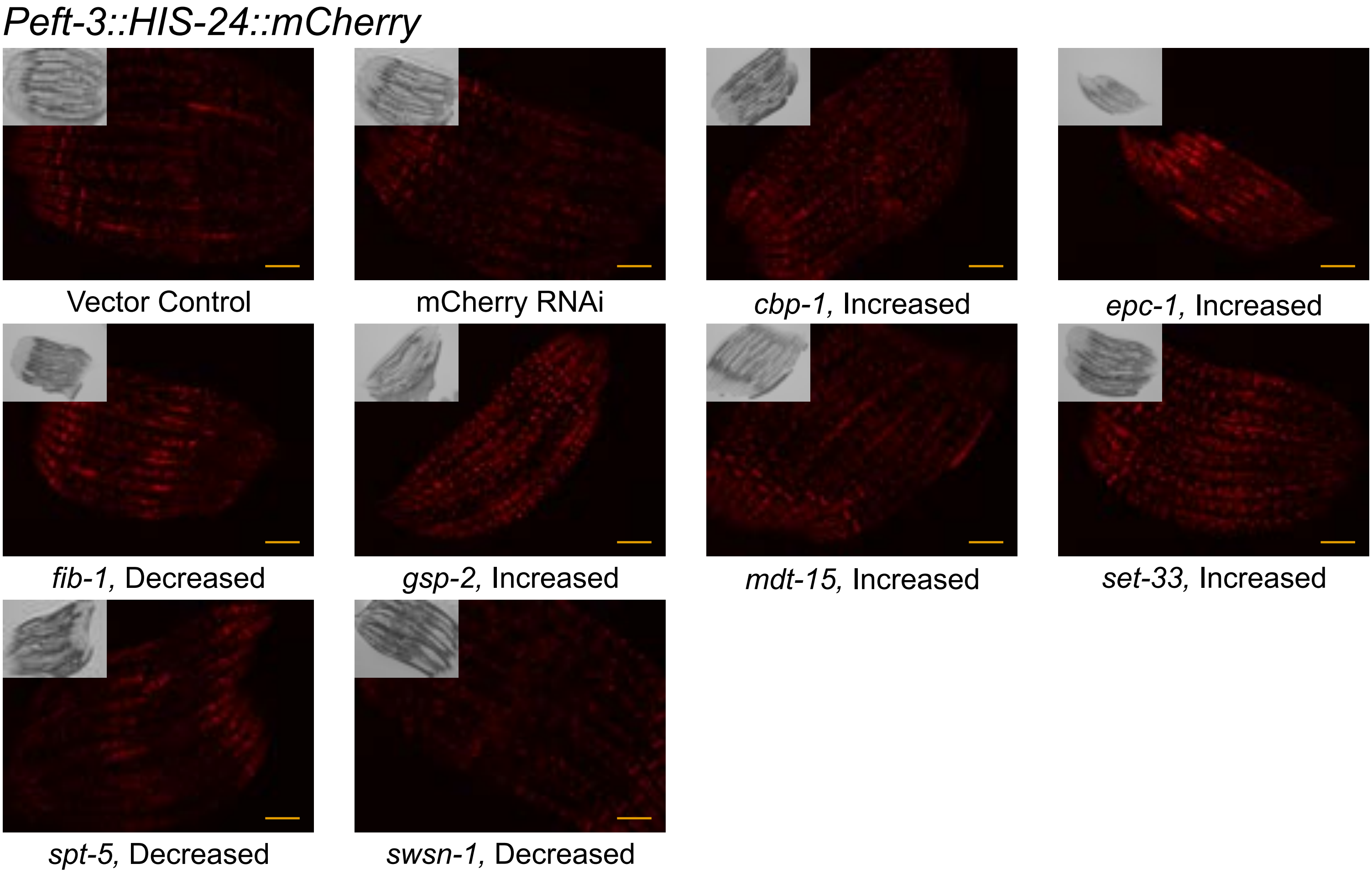

Peft-3::HIS-24::mCherry
Vector Control
mCherry RNAi
cbp-1, Increased
epc-1, Increased
fib-1, Decreased
gsp-2, Increased
mdt-15, Increased
set-33, Increased
spt-5, Decreased
swsn-1, Decreased

Supplemental Figure S5. RNAi interactions between CFs and *Peft-3::HIS-24::mCherry.* Scale bar=100μM.

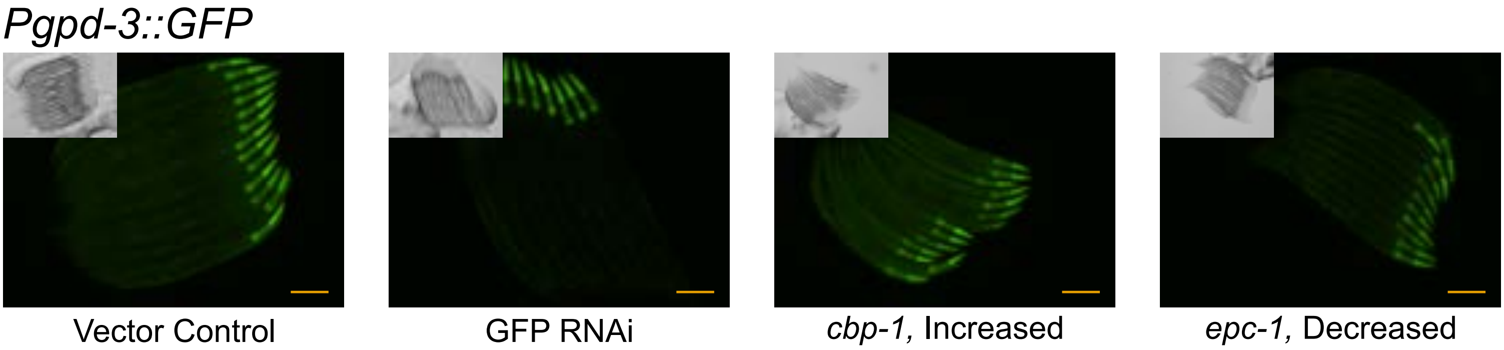
Pgpd-3::GFP
Vector Control
GFP RNAi
cbp-1, Increased
epc-1, Decreased

Supplemental Figure S6. RNAi interactions between CFs and *Pgpd-3::GFP.* Scale bar=100μM.

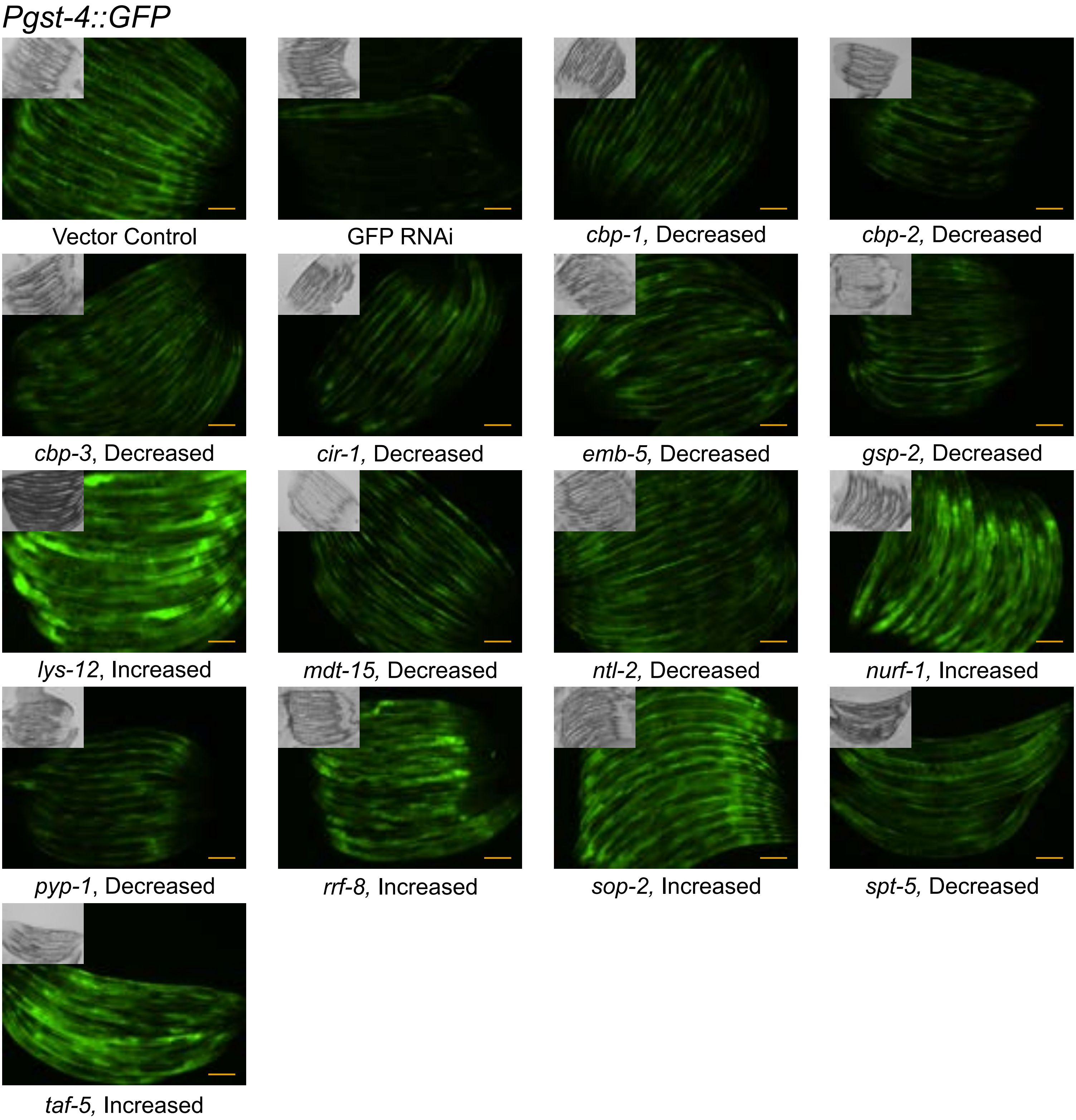

Pgst-4::GFP
Vector Control
GFP RNAi
cbp-1, Decreased
cbp-2, Decreased
cbp-3, Decreased
cir-1, Decreased
emb-5, Decreased
gsp-2, Decreased
lys-12, Increased
mdt-15, Decreased
ntl-2, Decreased
nurf-1, Increased
pyp-1, Decreased
rrf-8, Increased
sop-2, Increased
spt-5, Decreased
taf-5, Increased

Supplemental Figure S7. RNAi interactions between CFs and *Pgst-4::GFP.* Scale bar=100μM.

Phsp-3::HIS-24::mCherry

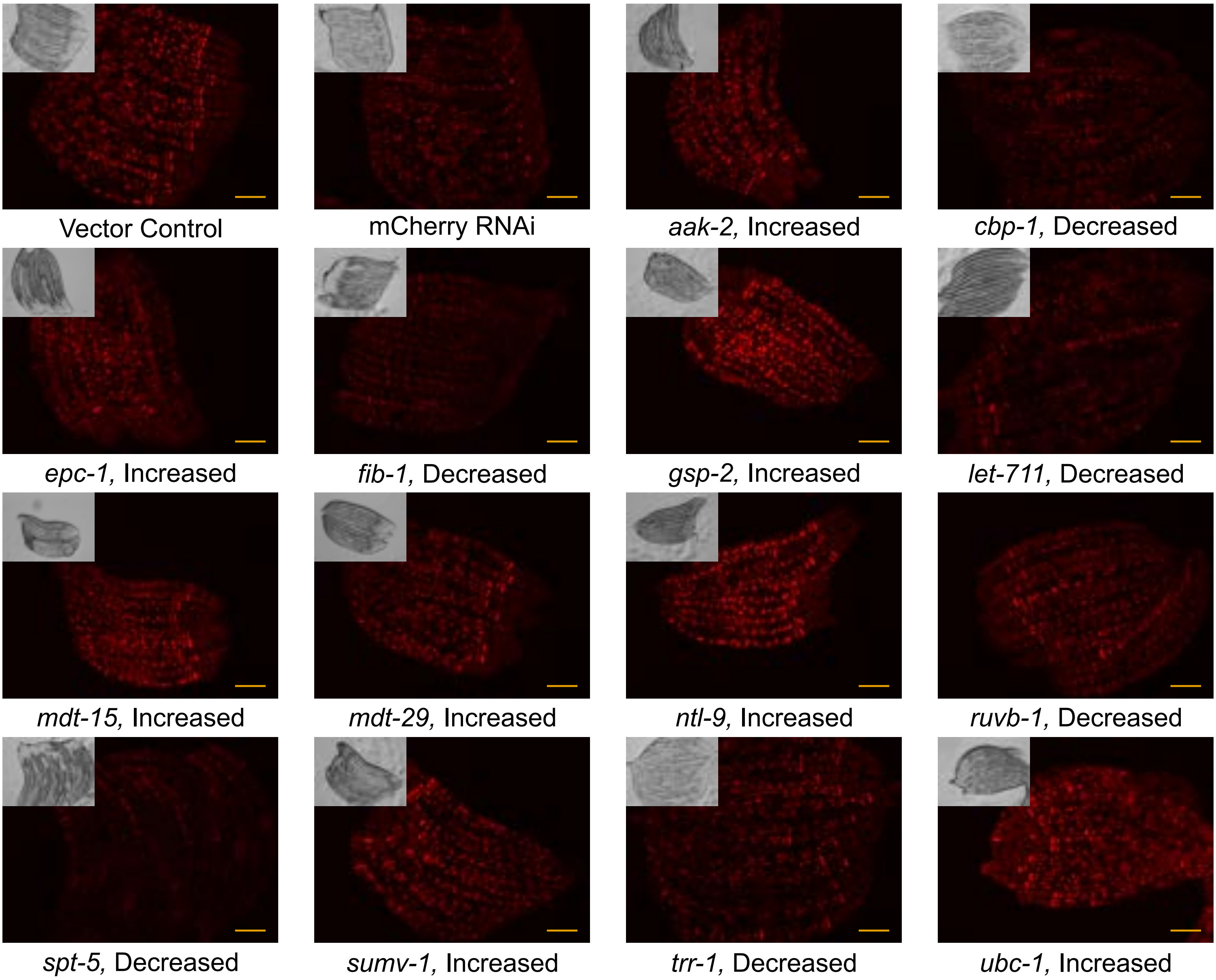

Supplemental Figure S8. RNAi interactions between CFs and *Phsp-3::HIS-24::mCherry.* Scale bar=100μM.

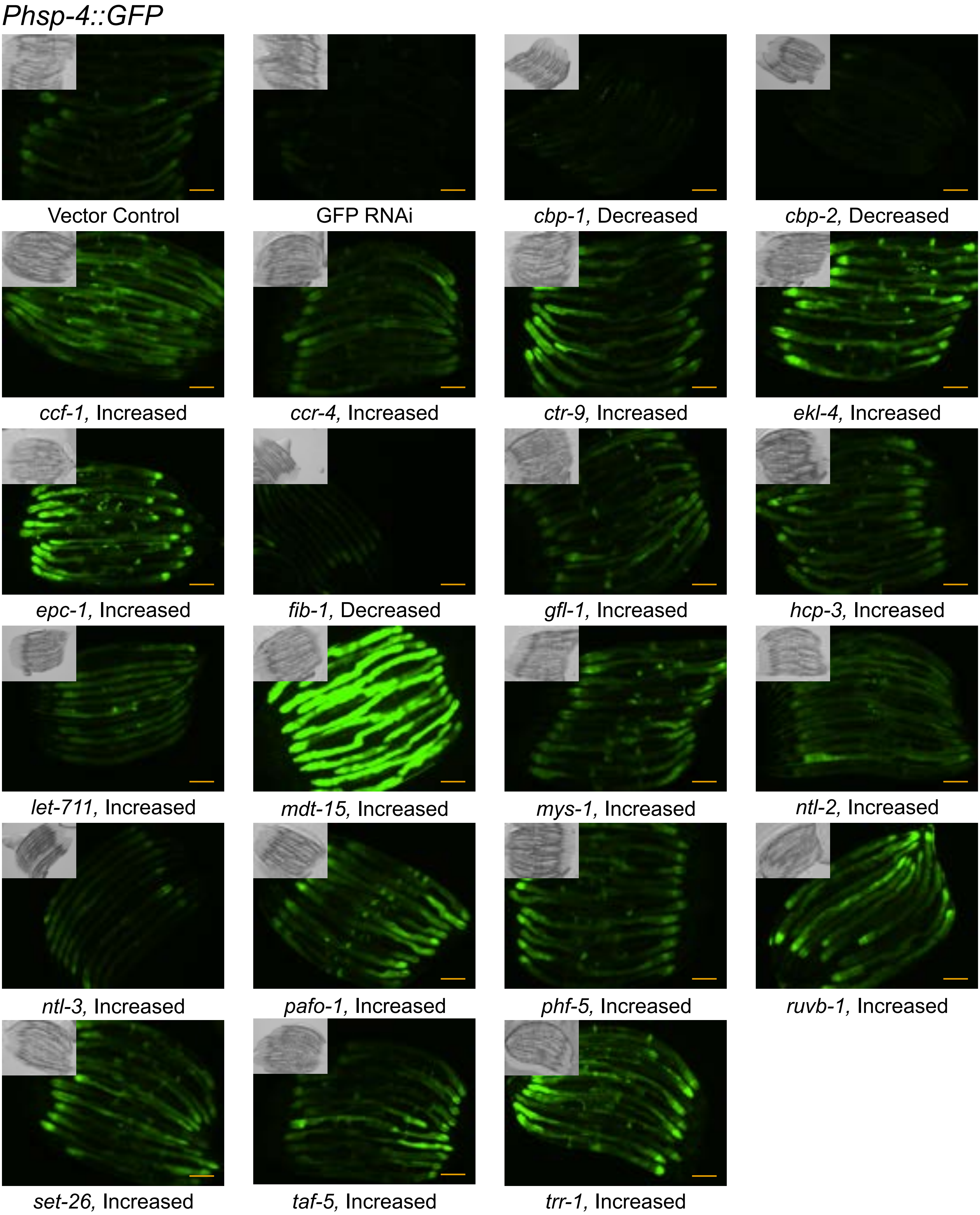

Phsp-4::GFP
Vector Control
GFP RNAi
cbp-1, Decreased
cbp-2, Decreased
ccf-1, Increased
ccr-4, Increased
ctr-9, Increased
ekl-4, Increased
epc-1, Increased
fib-1, Decreased
gfl-1, Increased
hcp-3, Increased
let-711, Increased
mdt-15, Increased
mys-1, Increased
ntl-2, Increased
ntl-3, Increased
pafo-1, Increased
phf-5, Increased
ruvb-1, Increased
set-26, Increased
taf-5, Increased
trr-1, Increased

Supplemental Figure S9. RNAi interactions between CFs and *Phsp-4::GFP.* Scale bar=100μM.

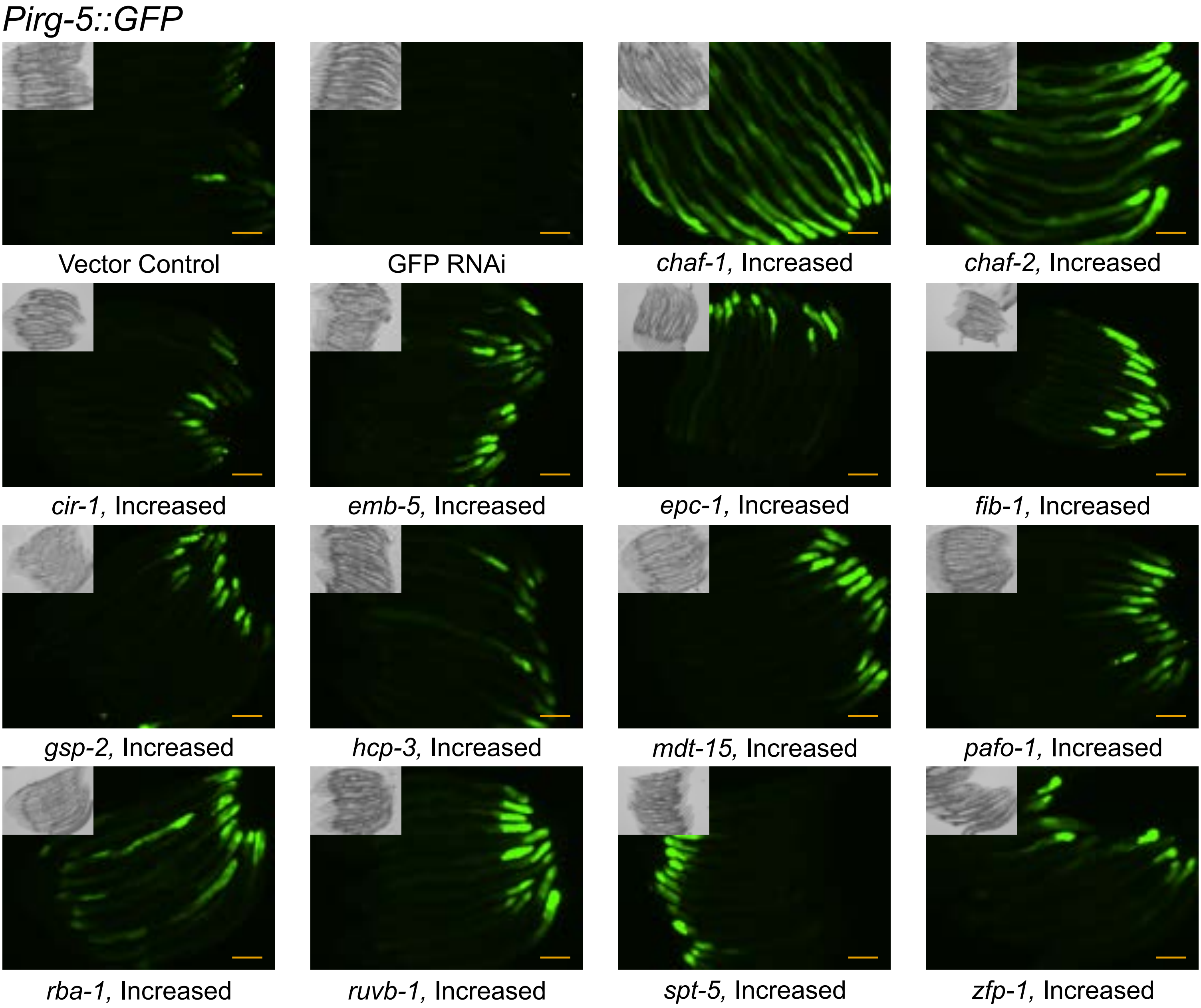

Pirg-5::GFP
Vector Control
GFP RNAi
chaf-1, Increased
chaf-2, Increased
cir-1, Increased
emb-5, Increased
epc-1, Increased
fib-1, Increased
gsp-2, Increased
hcp-3, Increased
mdt-15, Increased
pafo-1, Increased
rba-1, Increased
ruvb-1, Increased
spt-5, Increased
zfp-1, Increased

Supplemental Figure S10. RNAi interactions between CFs and *Pirg-5::GFP.* Scale bar=100µM.

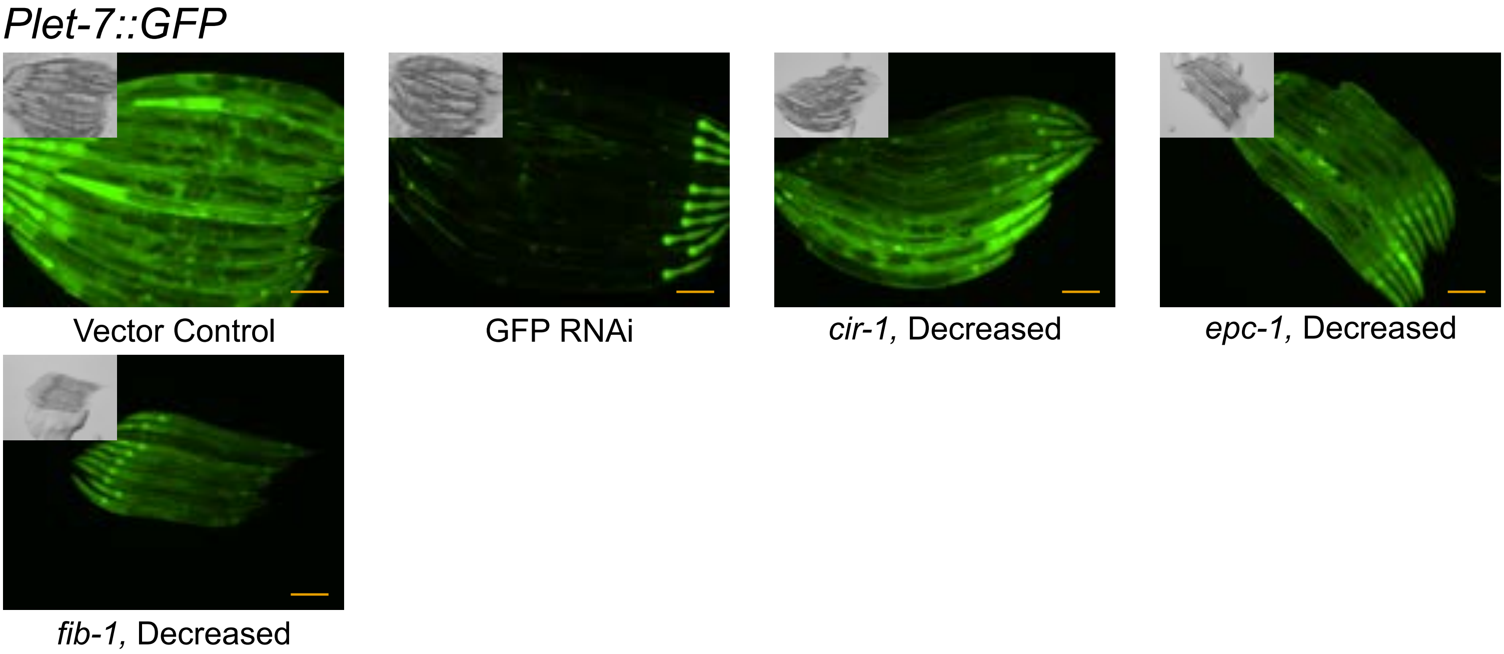
Plet-7::GFP
Vector Control
GFP RNAi
cir-1, Decreased
epc-1, Decreased
fib-1, Decreased

Supplemental Figure S11. RNAi interactions between CFs and *Plet-7::GFP.* Scale bar=100μM.

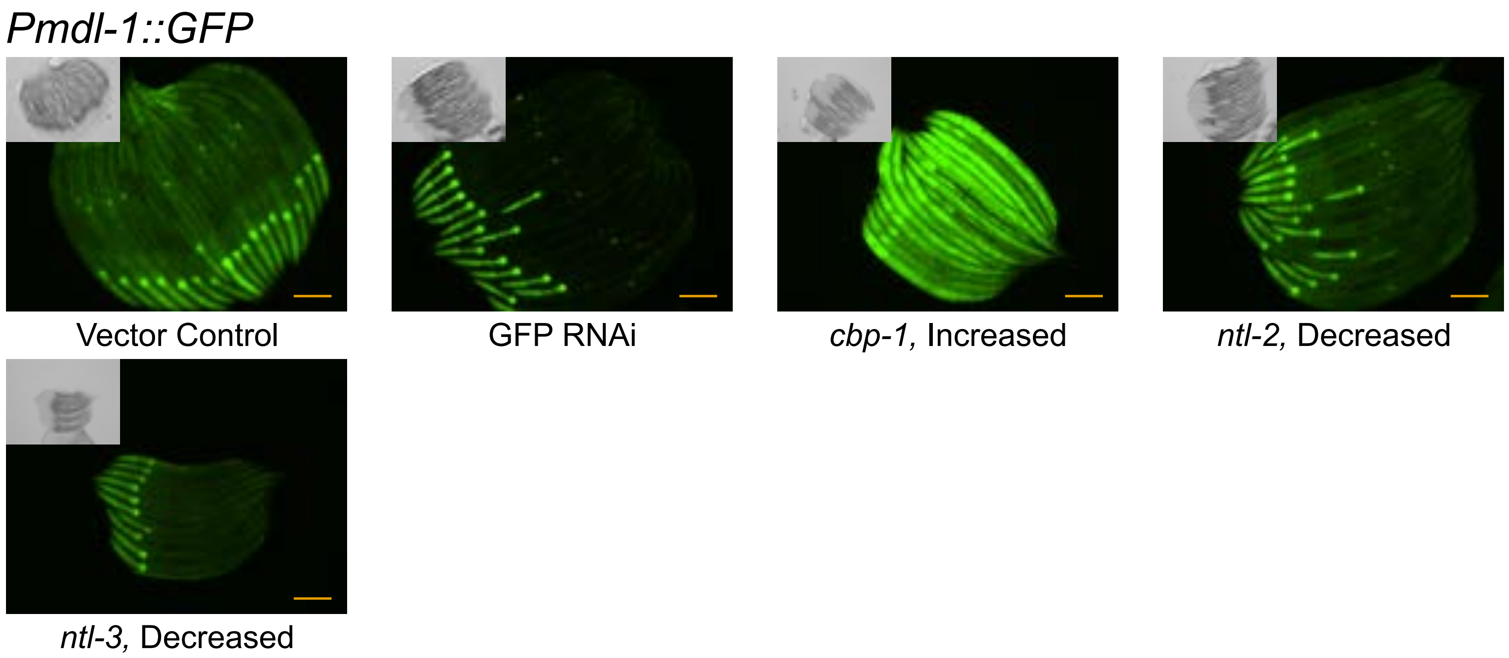

Pmdl-1::GFP
Vector Control
GFP RNAi
cbp-1, Increased
ntl-2, Decreased
ntl-3, Decreased

Supplemental Figure S12. RNAi interactions between CFs and *Pmdl-1::GFP.* Scale bar=100μM.

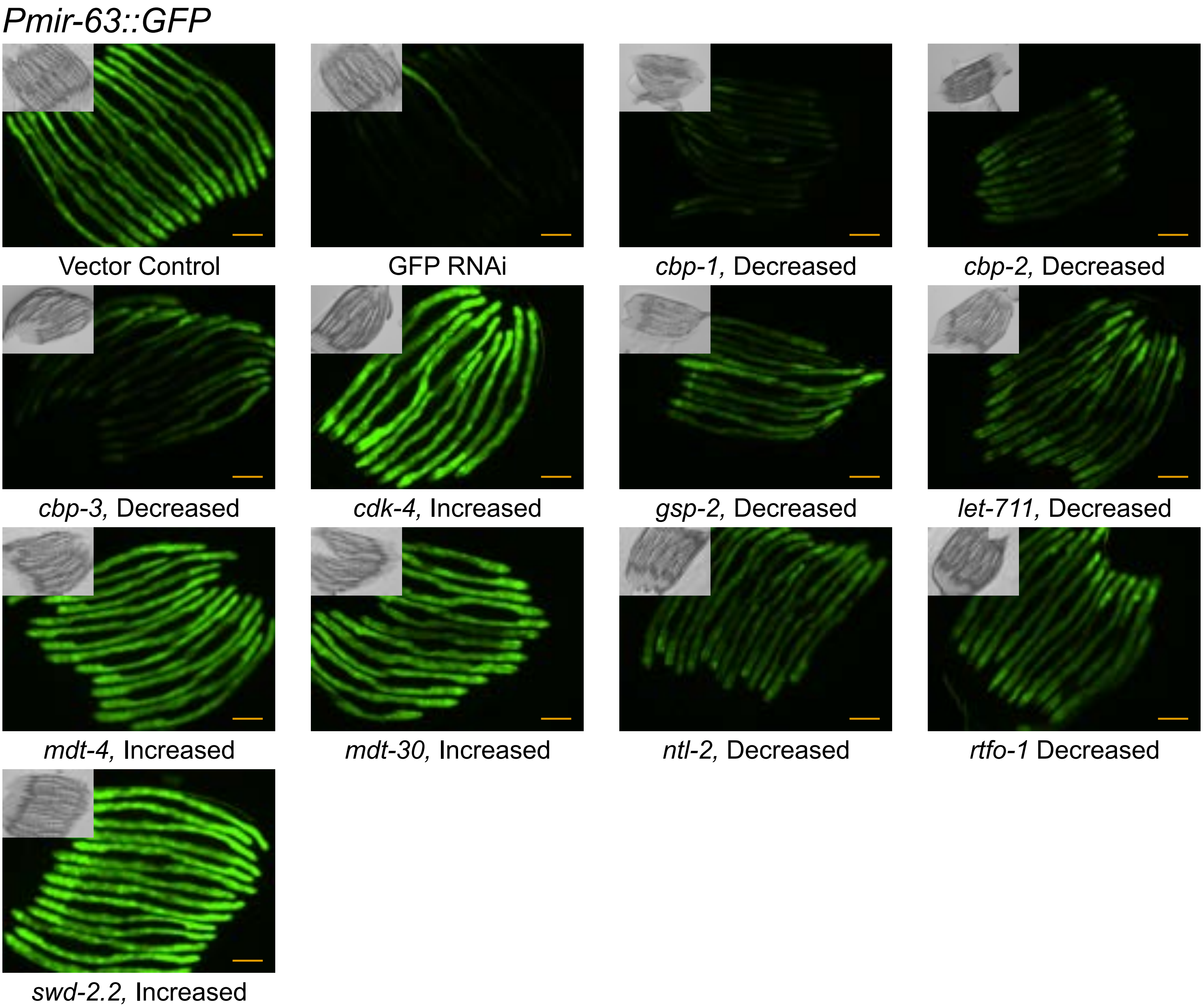
Pmir-63::GFP
Vector Control
GFP RNAi
cbp-1, Decreased
cbp-2, Decreased
cbp-3, Decreased
cdk-4, Increased
gsp-2, Decreased
let-711, Decreased
mdt-4, Increased
mdt-30, Increased
ntl-2, Decreased
rtfo-1 Decreased
swd-2.2, Increased

Supplemental Figure S13. RNAi interactions between CFs and *Pmir-63::GFP.*
Scale bar=100μM.

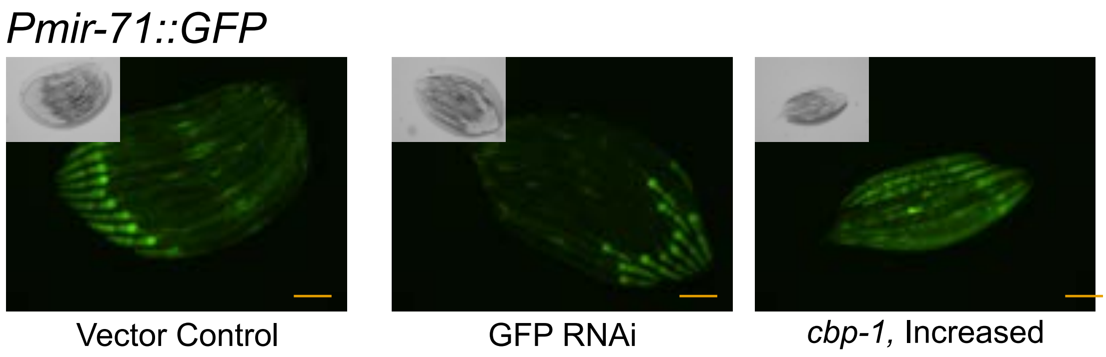
Pmir-71::GFP
Vector Control
GFP RNAi
cbp-1, Increased

Supplemental Figure S14. RNAi interactions between CFs and *Pmir-71::GFP.*
Scale bar=100μM.

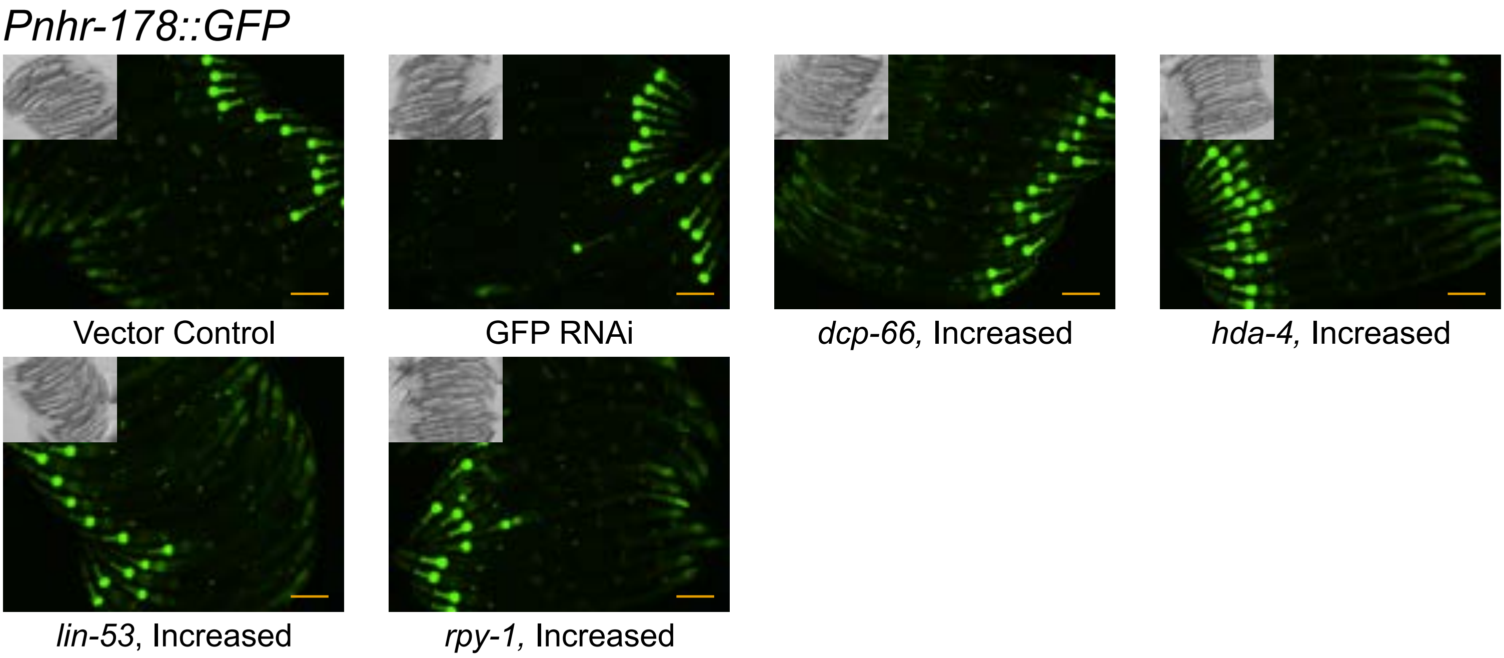

Pnhr-178::GFP
Vector Control
GFP RNAi
dcp-66, Increased
hda-4, Increased
lin-53, Increased
rpy-1, Increased

Supplemental Figure S15. RNAi interactions between CFs and *Pnhr-178::GFP.*
Scale bar=100µM.

Psbp-1::GFP

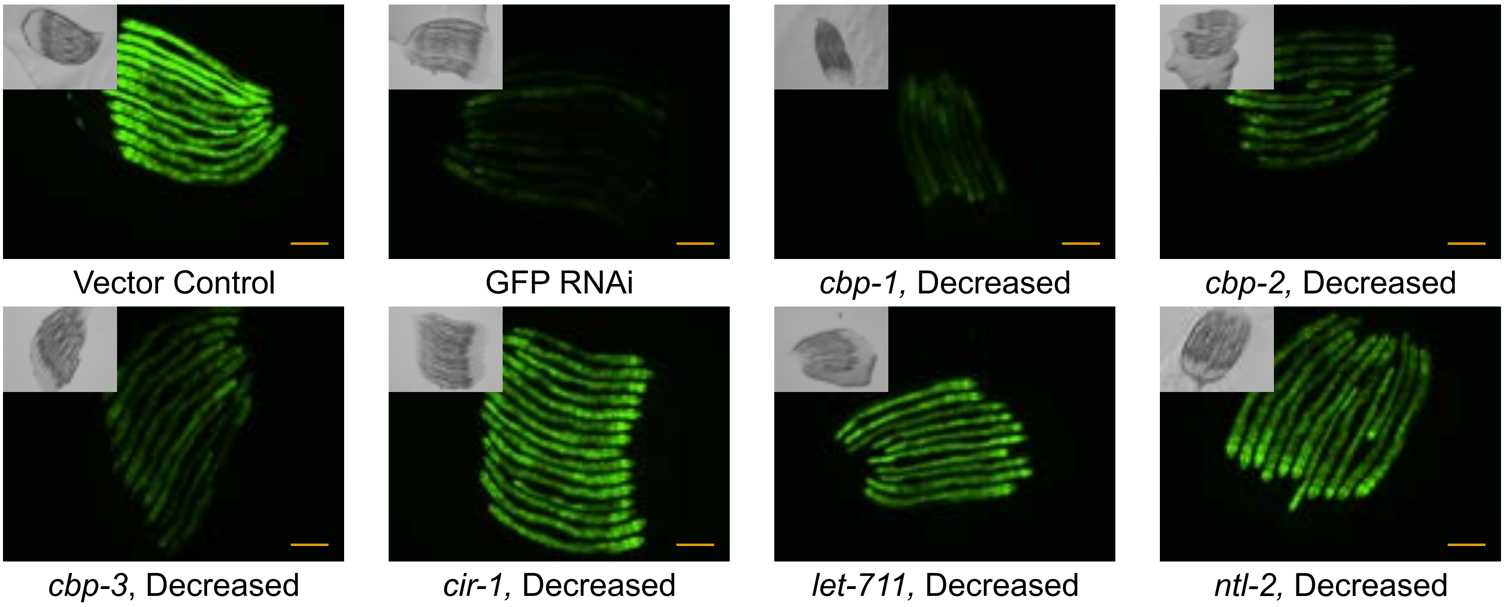

Vector Control
GFP RNAi
cbp-1, Decreased
cbp-2, Decreased
cbp-3, Decreased
cir-1, Decreased
let-711, Decreased
ntl-2, Decreased

Supplemental Figure S16. RNAi interactions between CFs and *Psbp-1::GFP.* Scale bar=100μM.

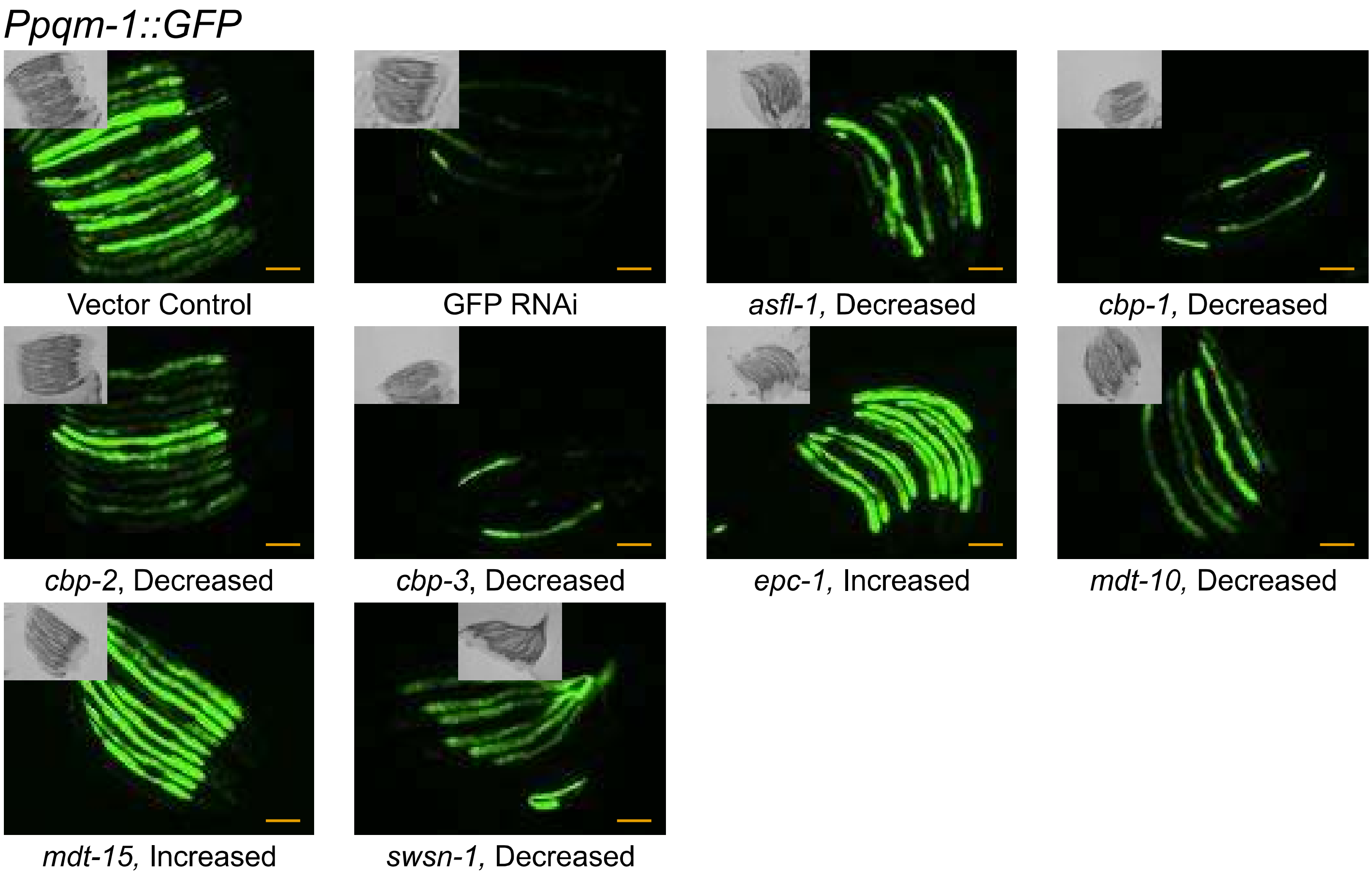

Ppqm-1::GFP
Vector Control
GFP RNAi
asfl-1, Decreased
cbp-1, Decreased
cbp-2, Decreased
cbp-3, Decreased
epc-1, Increased
mdt-10, Decreased
mdt-15, Increased
swsn-1, Decreased

Supplemental Figure S17. RNAi interactions between CFs and *Ppqm-1::GFP.*
Scale bar=100μM.

Psod-3::GFP

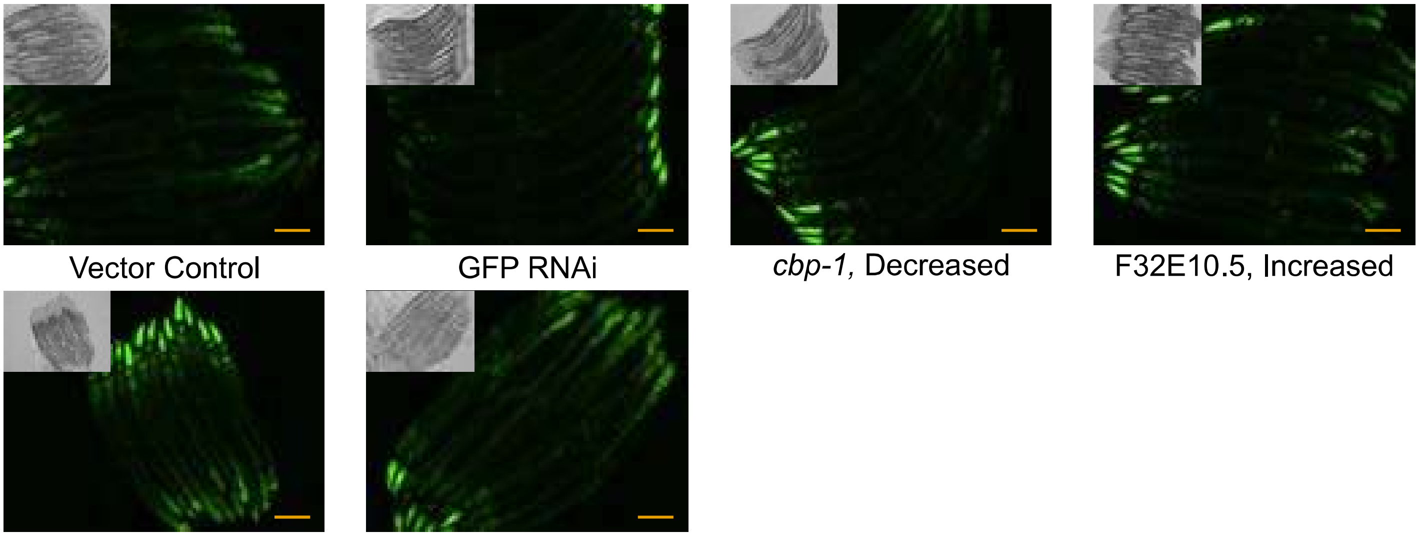

Vector Control

GFP RNAi

cbp-1, Decreased

F32E10.5, Increased

fib-1, Increased

mdt-15, Increased

Supplemental Figure S18. RNAi interactions between CFs and *Psod-3::GFP.* Scale bar=100μM.

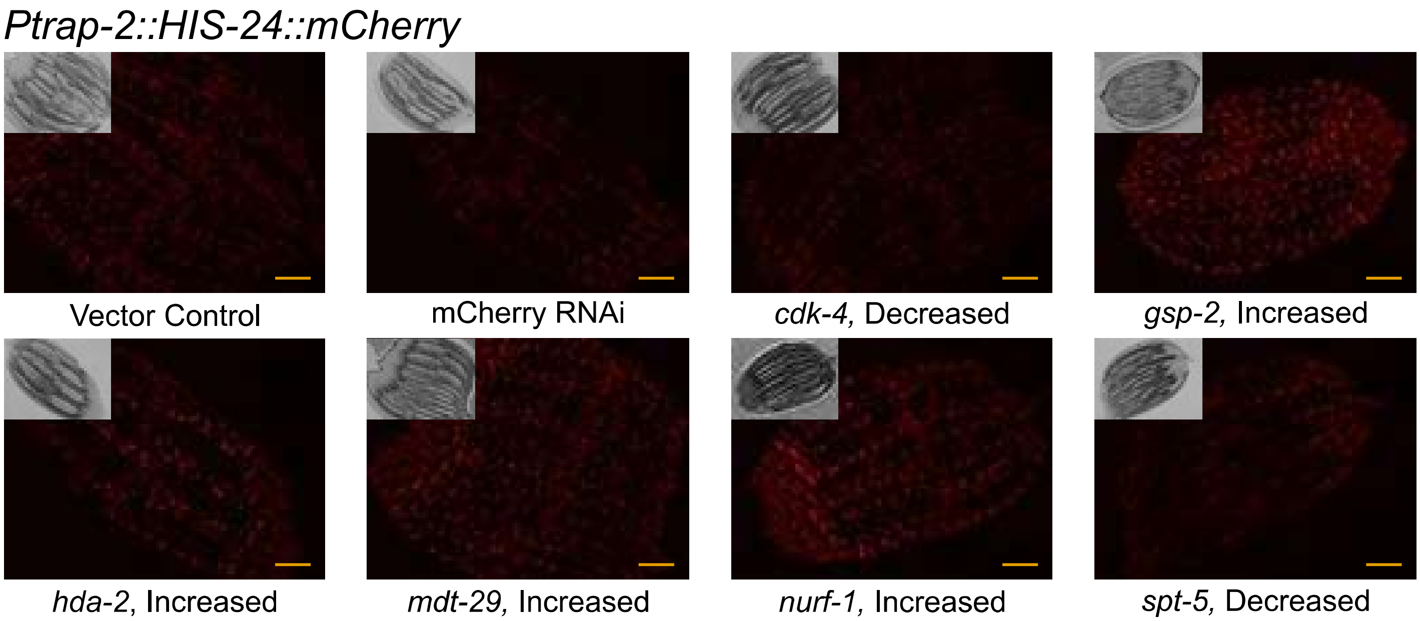
Ptrap-2::HIS-24::mCherry
Vector Control
mCherry RNAi
cdk-4, Decreased
gsp-2, Increased
hda-2, Increased
mdt-29, Increased
nurf-1, Increased
spt-5, Decreased

Supplemental Figure S19. RNAi interactions between CFs and *Ptrap-2::HIS-24:mCherry.* Scale bar=100μM.

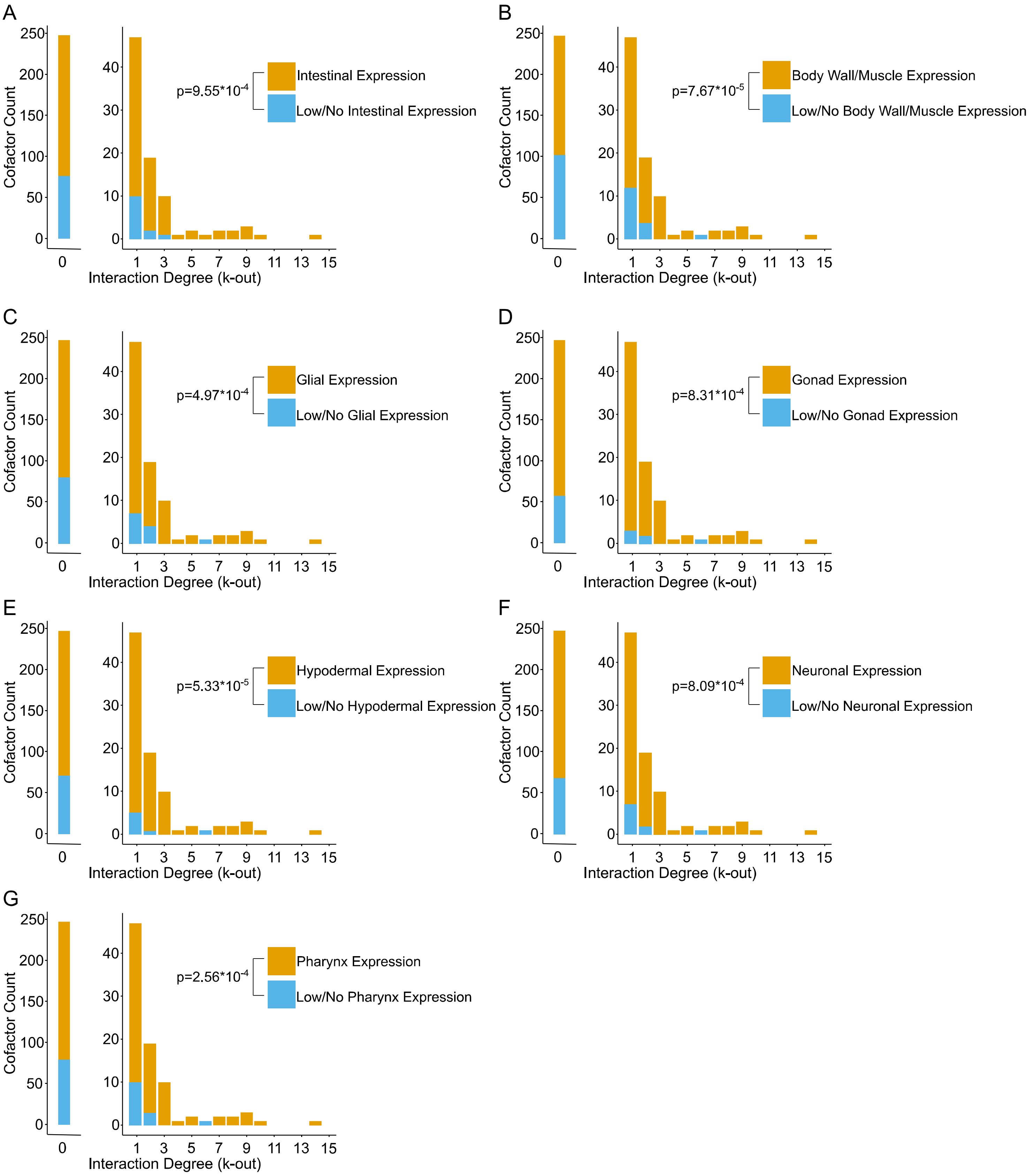
A
Cofactor Count
Interaction Degree (k-out)
$p=9.55*10^{-4}$
Intestinal Expression
Low/No Intestinal Expression
B
Cofactor Count
Interaction Degree (k-out)
$p=7.67*10^{-5}$
Body Wall/Muscle Expression
Low/No Body Wall/Muscle Expression
C
Cofactor Count
Interaction Degree (k-out)
$p=4.97*10^{-4}$
Glial Expression
Low/No Glial Expression
D
Cofactor Count
Interaction Degree (k-out)
$p=8.31*10^{-4}$
Gonad Expression
Low/No Gonad Expression
E
Cofactor Count
Interaction Degree (k-out)
$p=5.33*10^{-5}$
Hypodermal Expression
Low/No Hypodermal Expression
F
Cofactor Count
Interaction Degree (k-out)
$p=8.09*10^{-4}$
Neuronal Expression
Low/No Neuronal Expression
G
Cofactor Count
Interaction Degree (k-out)
$p=2.56*10^{-4}$
Pharynx Expression
Low/No Pharynx Expression

Supplemental Figure S20. Comparisons of k-out and tissue expression.

A-G: The k-out distribution, as in **Figure 4B.** Orange bars represent genes expressed in the following tissues: A: Intestine; B: Body Wall/Muscle; C: Glia; D: Gonad; E: Hypodermis; F: Neurons; G: Pharynx. Blue bars represent genes not expressed in those tissues. P-values were calculated using Mann-Whitney tests.

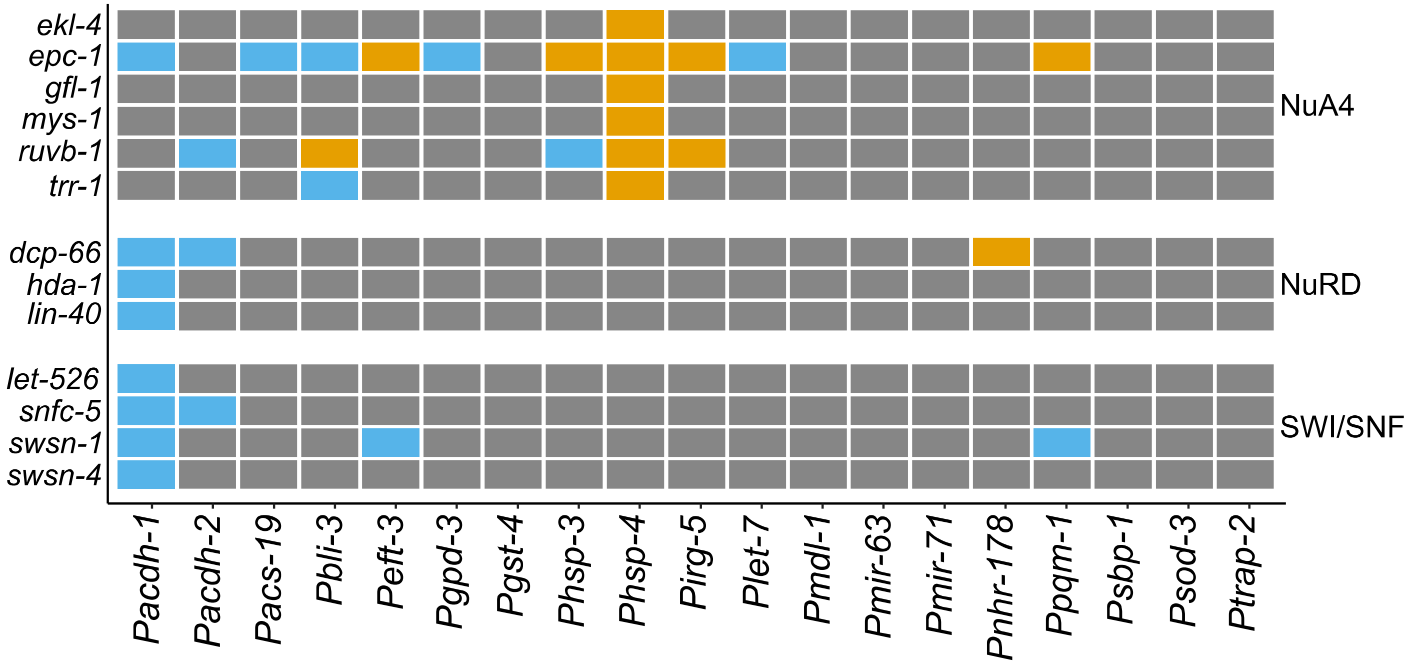
ekl-4
epc-1
gfl-1
mys-1
ruvb-1
trr-1
NuA4
dcp-66
hda-1
lin-40
NuRD
let-526
snfc-5
swsn-1
swsn-4
SWI/SNF
Pacdh-1
Pacdh-2
Pacs-19
Pbli-3
Peft-3
Pgpd-3
Pgst-4
Phsp-3
Phsp-4
Pirg-5
Plet-7
Pmdl-1
Pmir-63
Pmir-71
Pnhr-178
Ppqm-1
Psbp-1
Psod-3
Ptrap-2

Supplemental Figure S21. Additional CF complex interaction profiles

Interaction profiles for NuA4, NuRD, and SWI/SNF complexes. Orange boxes represent increases in fluorescence (repressing interactions) and blue boxes represent decreases in fluorescence (activating interactions). Complex components with no interactions were excluded from the figure.

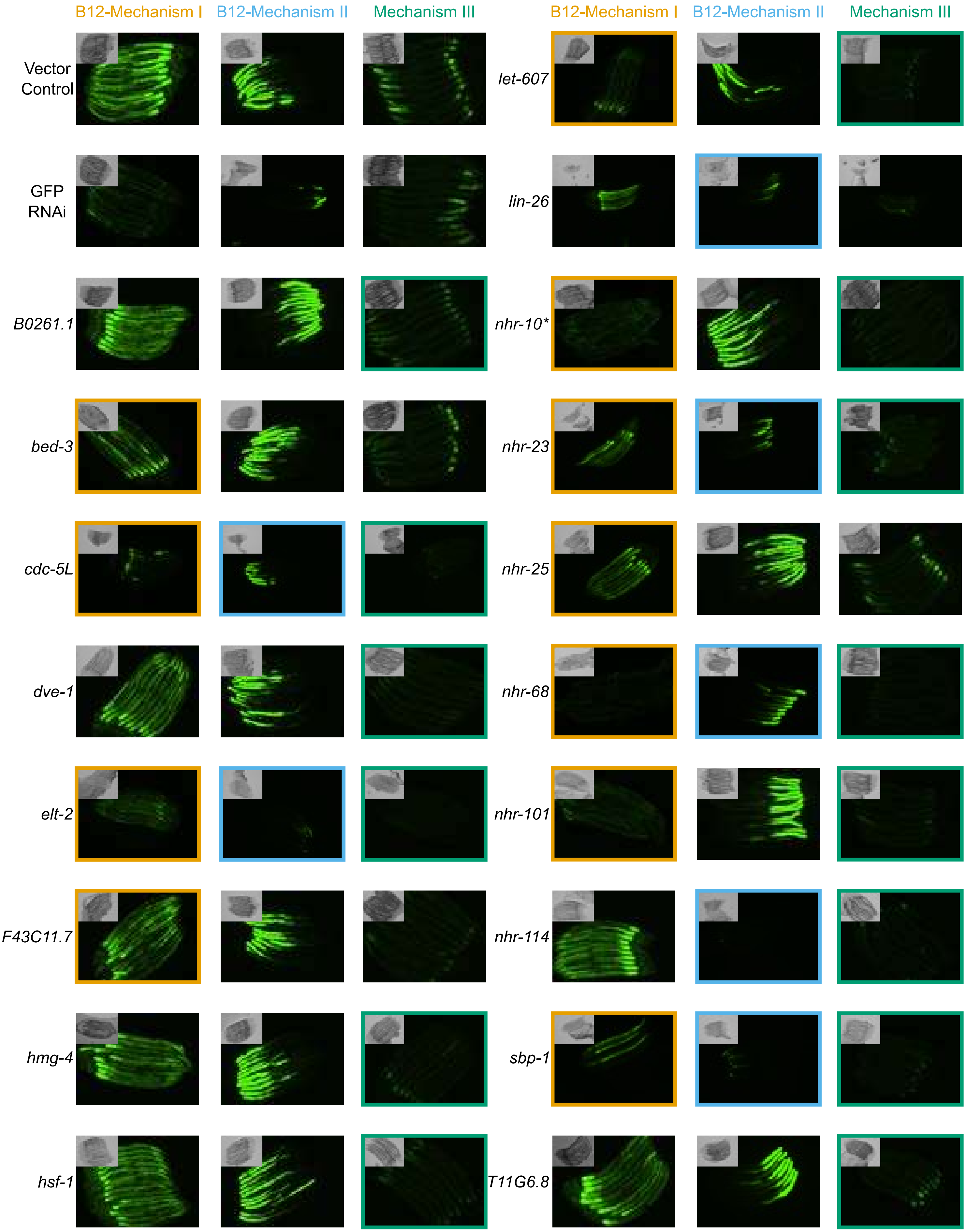

B12-Mechanism I
B12-Mechanism II
Mechanism III
B12-Mechanism I
B12-Mechanism II
Mechanism III
Vector Control
GFP RNAi
B0261.1
bed-3
cdc-5L
dve-1
elt-2
F43C11.7
hmg-4
hsf-1
let-607
lin-26
nhr-10*
nhr-23
nhr-25
nhr-68
nhr-101
nhr-114
sbp-1
T11G6.8

Supplemental Figure S22. TF interactions with *Pacdh-1::GFP* activation mechanisms

Images of *Pacdh-1::GFP* under the three activation mechanisms with each TF RNAi that interacts with at least one activation mechanism. TFs with no interactions are not shown. Borders indicate an interaction.

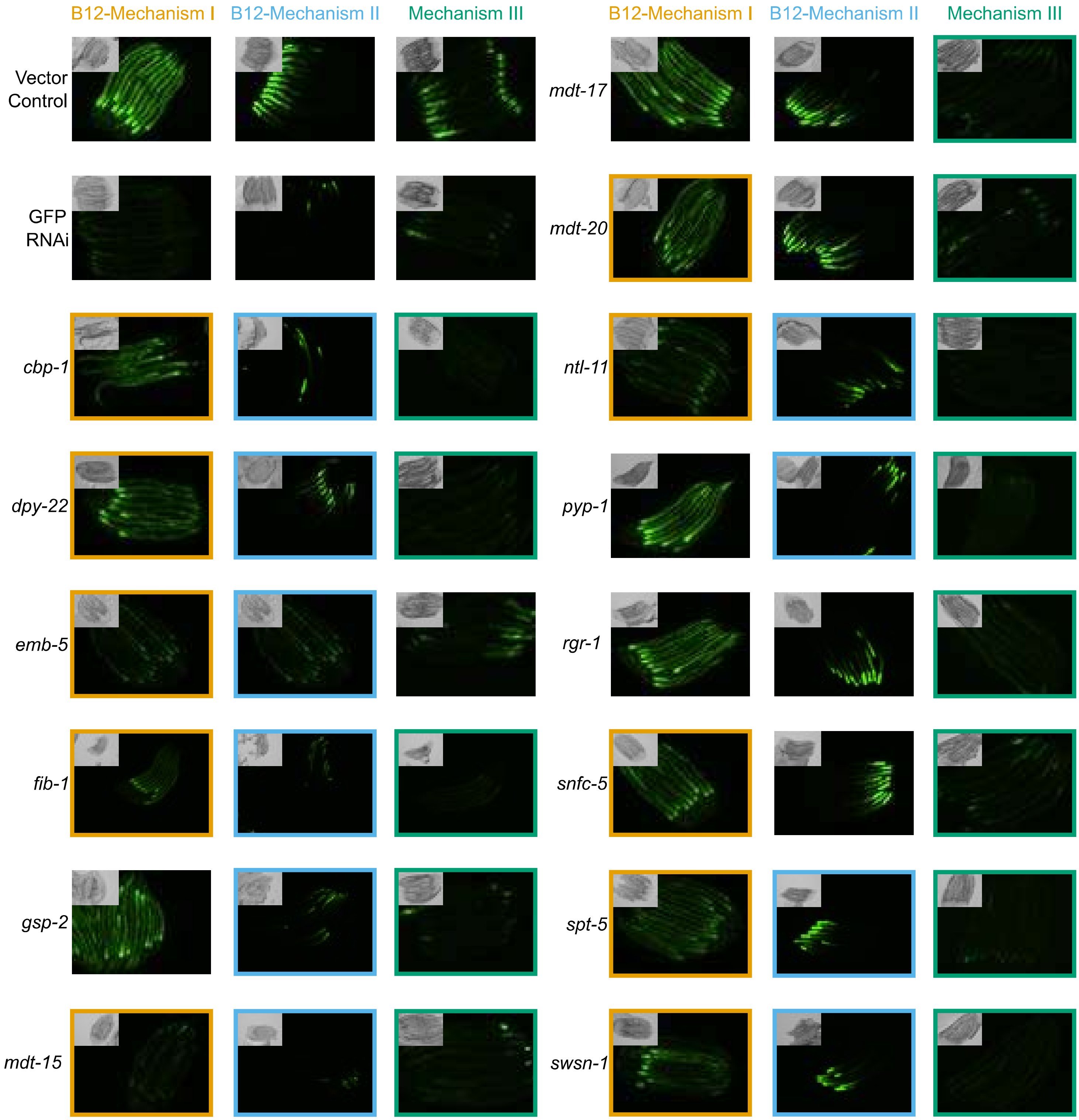

B12-Mechanism I
B12-Mechanism II
Mechanism III
B12-Mechanism I
B12-Mechanism II
Mechanism III
Vector Control
GFP RNAi
cbp-1
dpy-22
emb-5
fib-1
gsp-2
mdt-15
mdt-17
mdt-20
ntl-11
pyp-1
rgr-1
snfc-5
spt-5
swsn-1

Supplemental Figure S23. CF interactions with *Pacdh-1::GFP* activation mechanisms

Images of *Pacdh-1::GFP* under the three activation mechanisms with each CF RNAi that interacts with at least one activation mechanism. CFs with no interactions are not shown. Borders indicate an interaction.
