# Supplementary material for: A transcriptional cofactor regulatory network for the C. elegans intestine
Source: G3 (Bethesda). 2023 Apr 29;13(7):jkad096. doi: 10.1093/g3journal/jkad096 (PMC10320766; doi:10.1093/g3journal/jkad096)
Supplement: jkad096_Supplementary_Data [file jkad096_supplementary_data.zip › Supplemental_Material_Legends_G3-2023-404230.docx]

**File S1.** Supplemental Figures S1-S22.

**Table S1.** CFs encoded in the *C. elegans* genome.

**Table S2.** Hypergeometric enrichments for essentiality among CF classes, CF binding domain-containing proteins, and for CFs, TFs, and metabolic genes.

**Table S3.** Description of *C. elegans* strains used in the primary screen.

**Table S4**. Interactions between CF RNAi strains and the 19 promoter reporters.

**Table S5.** Hypergeometric enrichments for interactions between CF classes or complexes and promoter reporters.

**Table S6.** Oligonucleotides used in this study.
